# Autoreactive T cell receptors with shared germline-like α chains in type 1 diabetes

**DOI:** 10.1172/jci.insight.151349

**Published:** 2021-11-22

**Authors:** Peter S. Linsley, Fariba Barahmand-pour-Whitman, Elisa Balmas, Hannah A. DeBerg, Kaitlin J. Flynn, Alex K. Hu, Mario G. Rosasco, Janice Chen, Colin O’Rourke, Elisavet Serti, Vivian H. Gersuk, Keshav Motwani, Howard R. Seay, Todd M. Brusko, William W. Kwok, Cate Speake, Carla J. Greenbaum, Gerald T. Nepom, Karen Cerosaletti

**Affiliations:** 1 Center for Systems Immunology,; 2 Center for Translational Research, and; 3 Center for Interventional Immunology, Benaroya Research Institute at Virginia Mason, Seattle, Washington, USA.; 4 Immune Tolerance Network, Bethesda, Maryland, USA.; 5 Department of Pathology, Immunology, and Laboratory Medicine, College of Medicine, University of Florida, Gainesville, Florida, USA.; 6 University of Florida Diabetes Institute, University of Florida, Gainesville, Florida, USA.; 7 FlowJo, LLC, Ashland, Oregon, USA.; 8 Department of Pediatrics, College of Medicine, University of Florida, Gainesville, Florida, USA.

**Keywords:** Autoimmunity, T cell receptor

## Abstract

Human islet antigen reactive CD4^+^ memory T cells (IAR T cells) play a key role in the pathogenesis of autoimmune type 1 diabetes (T1D). Using single-cell RNA sequencing (scRNA-Seq) to identify T cell receptors (TCRs) in IAR T cells, we have identified a class of TCRs that share TCRα chains between individuals (“public” chains). We isolated IAR T cells from blood of healthy, new-onset T1D and established T1D donors using multiplexed CD154 enrichment and identified paired TCRαβ sequences from 2767 individual cells. More than a quarter of cells shared TCR junctions between 2 or more cells (“expanded”), and 29/47 (~62%) of expanded TCRs tested showed specificity for islet antigen epitopes. Public TCRs sharing TCRα junctions were most prominent in new-onset T1D. Public TCR sequences were more germline like than expanded unique, or “private,” TCRs, and had shorter junction sequences, suggestive of fewer random nucleotide insertions. Public TCRα junctions were often paired with mismatched TCRβ junctions in TCRs; remarkably, a subset of these TCRs exhibited cross-reactivity toward distinct islet antigen peptides. Our findings demonstrate a prevalent population of IAR T cells with diverse specificities determined by TCRs with restricted TCRα junctions and germline-constrained antigen recognition properties. Since these “innate-like” TCRs differ from previously described immunodominant TCRβ chains in autoimmunity, they have implications for fundamental studies of disease mechanisms. Self-reactive restricted TCRα chains and their associated epitopes should be considered in fundamental and translational investigations of TCRs in T1D.

## Graphical abstract

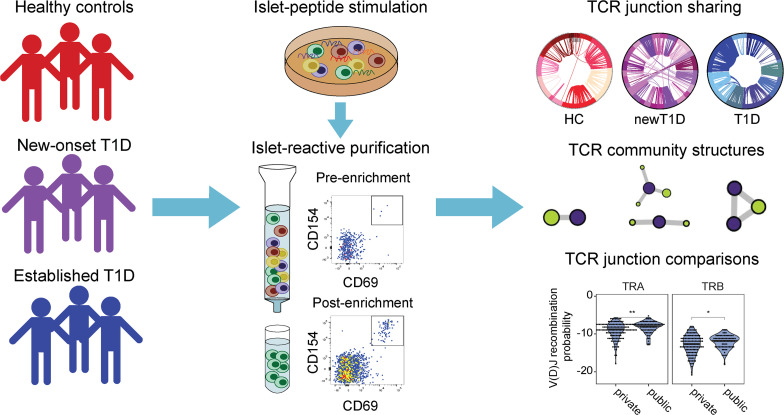

## Introduction

Human islet antigen reactive CD4^+^ memory T cells (IAR T cells) are widely studied for their role in β cell destruction and as therapeutic targets and biomarkers (1–6) of type 1 diabetes (T1D). Many efforts in humans have focused on IAR T cells in peripheral blood rather than the pancreas, which is not readily biopsied. IAR T cells can be detected at low frequency in blood of at-risk and T1D patients, as well as in healthy controls (HCs) (7–9).

A defining characteristic of all T cells is the ability of their T cell receptors (TCRs) to recognize antigenic peptides presented in the context of major histocompatibility (MHC) molecules. Each T cell expresses a distinct TCR clonotype, most commonly comprising TCRα and β chains (*TRA* and *TRB*, respectively) created by stochastic V(D)J recombination of germline encoded gene segments. This process has the potential to be extremely diverse, with a vast potential repertoire size (10, 11), which is further expanded because of random deletion and insertion of nucleotides at the recombination sites. T cells with TCRs recognizing foreign or self-antigens proliferate in response to recognition of antigenic peptides, resulting in clonal expansion of a population of cells with identical TCR sequence and antigen specificity (12). Expansion of cells with self-reactive clonotypes supports their role in driving and propagating disease in an autoimmune disease model (13).

While the extreme diversity of TCR sequences allows for private expanded TCR clonotypes, public expanded TCR sequences are sometimes observed (14). The dichotomy between public and private sequences is influenced by sampling depth and cohort size, suggesting that publicness is a continuous rather than a binary quantity (14). Shared public sequences are especially prominent in TCRs recognizing microbial antigens (15–17) and have been implicated in models of autoimmune disease (18–21), including T1D (22–24). Either *TRA* or *TRB* chains may be preferentially shared in different repertoires, with generally more sharing of the individual chains than with the paired *TRA*-*TRB* receptors (16). Dominant shared *TRB* chains have been linked to inducible autoimmunity in mouse models (18–21). Studies in mice identified a restricted set of abundant public TRB complementarity-determining region 3 sequences associated with self-reactivity (25). In contrast, in the nonobese diabetic (NOD) mouse model of T1D, diabetes is associated with restricted germline-like *TRA* chains targeting insulin (22–24). Less is known regarding public TCRs in human autoimmunity. In human T1D, self-reactive TCRs with both private (26) and public *TRB* sequences (27) have been described. Studies on TCRs as biomarkers in T1D have largely utilized *TRB* sequences (28). In contrast, CD8^+^ T cells reactive with the islet autoantigen islet-specific glucose-6-phosphatase catalytic subunit-related protein (IGRP) utilized restricted *TRA* chains (29). Public TCRs imply shared pathways to autoimmunity and therefore represent better candidate biomarkers and therapeutic targets (28).

Increasingly, phenotypic and TCR diversity of T cells are being explored by genome-wide single-cell RNA sequencing (scRNA-Seq) (26, 30–37). In a previous exploratory study combining flow cytometry–based assays and scRNA-Seq, we described methods to identify TCR sequences in parallel with full transcriptome phenotypes from individual IAR T cells (26). We focused on CD4^+^ T cells because of the strong genetic link between T1D and the HLA class II region, which regulates antigen presentation to CD4^+^ T cells. Strikingly, we observed predominantly private TCRs in IAR T cells from a limited set of 3 each healthy control (HC) and established T1D (T1D) donors. We also found extensive TCR clonotype sharing in IAR T cells from T1D subjects, consistent with in vivo T cell expansion during disease progression. Here we examined IAR T cells from a broader cohort of HC, new-onset T1D (newT1D), and established T1D patients. We demonstrate that public and private TCRs from IAR T cells have different properties and change in proportion as T1D progresses.

## Results

### Isolation and scRNA-Seq of IAR T cells from blood.

Our central hypothesis is that in vivo expansion of IAR T cells drives autoimmune destruction of the pancreas during T1D. This predicts that clonal populations important during disease progression share TCR sequences. To investigate the expansion of IAR T cells, we extended our previous comparisons of IAR T cells from T1D and HC patients (26). For the present studies, we expanded the number of participants analyzed to a total of 50 participants, including well-characterized T1D and matched HC participants, as well as a cohort of patients with new-onset T1D (newT1D) (38). Patient characteristics are summarized in Table 1 and presented in more detail in Supplemental Table 1; supplemental material available online with this article; https://doi.org/10.1172/jci.insight.151349DS1 Age did not differ significantly between groups (Table 1). T1D patients were tested at a median of about 3.6 years after diagnosis; newT1D patients were tested at less than 100 days from diagnosis. Importantly, about 90% of patients had high-risk DRB1*0401 HLA class II alleles, while about 10% had DRB1*0301 alleles.

Multiple islet epitopes related to T1D have been identified (39). To screen for epitopes from several disease-relevant proteins, including glutamic acid decarboxylase 65 kDa isoform (GAD65), preproinsulin, IGRP, zinc transporter 8 (ZNT8), and tyrosine phosphatase-related islet antigen 2 (IA-2), in a side-by-side manner, we used a multiplexed CD154 enrichment procedure (40). This procedure identifies CD4^+^ T cells stimulated to express the activation marker CD154 upon treatment of banked PBMCs with pools of class II–restricted islet antigen peptides (Supplemental Table 2 and Supplemental Figure 1A). We utilized 2 peptide pools: a pool of 28 peptides for HC and T1D, all of whom were DRB1*0401 patients (0401 pool), and another pool for newT1D patients, containing 7 additional peptides to accommodate the DRB1*0301 patients (0401/0301/DQ8 pool). The peptides we used represent consensus immunodominant epitopes recognized by CD4^+^ T cells in DRB1*0401, DRB1*0301, and *DQ8* T1D patients over many published (26, 41–43) and unpublished epitope mapping studies. From our previous scRNA-Seq study (26), we expected that most T1D patients would have cells reactive with some, but not all, of the peptides used.

Following peptide stimulation, cells with upregulated CD154 were enriched using magnetic bead separation (Supplemental Figure 1A), then sorted for cells that had coexpressed the CD154 and CD69 activation markers (Supplemental Figure 1B). For scRNA-Seq, we initially sorted cells into microfluidic chips (26) but later transitioned to 96-well plate sorts (Supplemental Figure 1). We found plate-sorted cells gave better yields of high-quality profiles and enabled the use of index sorting to determine cell surface phenotypes of each single sorted cell. We sorted mainly memory (CD45RA^–^, CD45RO^+^) (Supplemental Figure 1B), though in some cases we sorted total IAR T cells (CD45RA^+^ plus CD45RO^+^), to increase cell yield. Unless noted otherwise, analyses reported here utilized memory cells because they are antigen experienced and more likely involved in ongoing autoimmunity.

Sorted cells that were subjected to scRNA-Seq and RNA-Seq reads were processed to identify rearranged TCR chains (Supplemental Methods). After initial quality control filtering, we identified TCRs in *n* = 2767 cells (profiles) from individual IAR T cells (457, 1489, and 821 cells from HC, newT1D, and T1D, respectively). This corresponds to means of 38, 62, and 68 profiles/person from each disease group. By amino acid sequences, these cells expressed 4296 unique TCR junctions, corresponding to 2160 and 2136 *TRA* and *TRB* junctions, respectively. These TCR sequences are listed in Supplemental Table 3 (Total TCRs). A subset of 741 cells shared junctions with other cells (741/2767, ~27% of total cells) (expanded cells). In some cases, TCRs were subjected to additional filtering before use in subsequent analyses (Supplemental Methods).

### Expanded IAR T cells recognize multiple islet epitopes.

**Figure 1:**
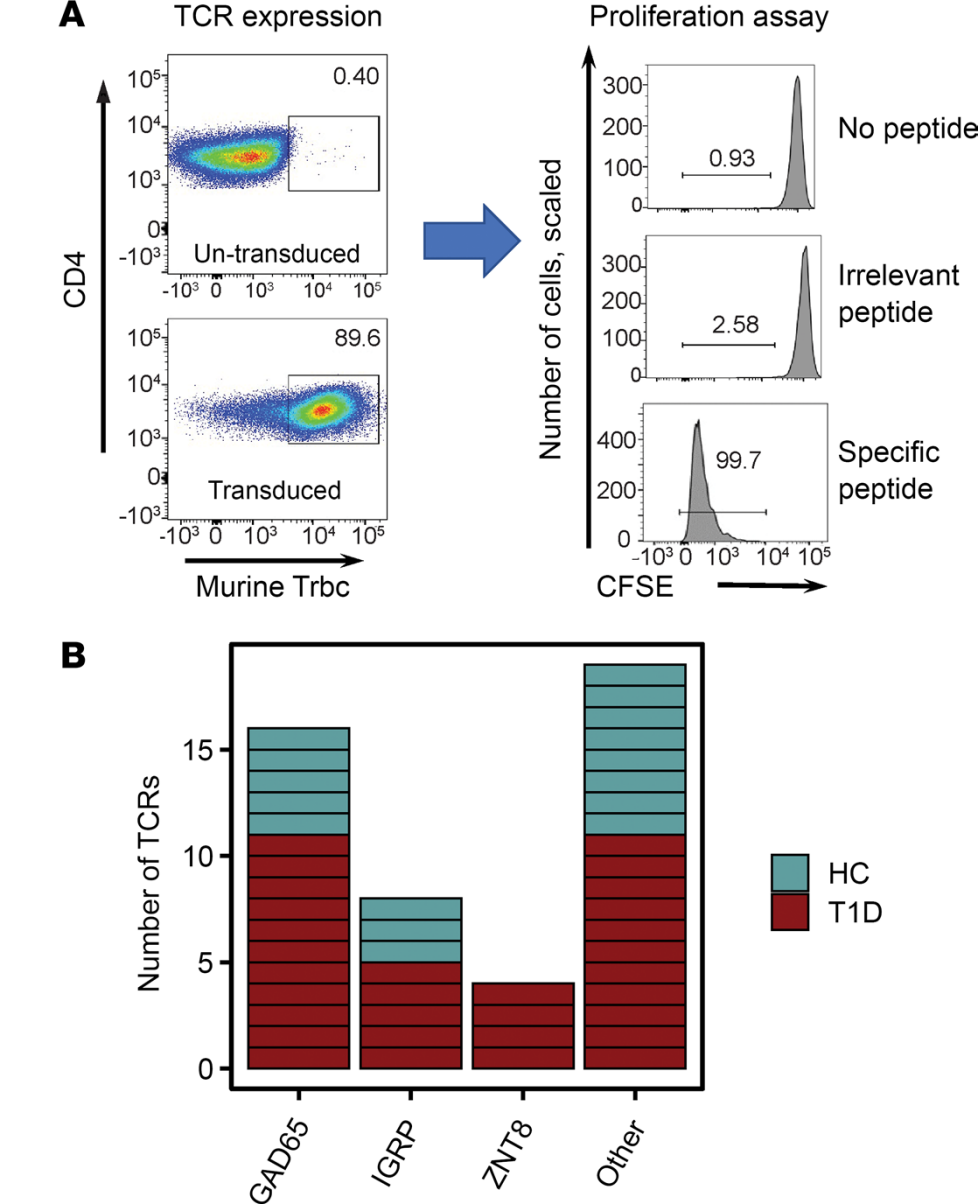
Expanded IAR T cell TCRs identified by scRNA-Seq recognize multiple epitopes. (**A**) Expression and functional activity of recombinant TCRs following lentiviral transduction. Primary human CD4^+^ T cells were transduced with recombinant *TRA* and *TRB* chains cloned in a lentiviral vector upstream of the murine *Trac* and *Trbc* constant region gene segments, respectively. Transduced cells were identified by flow cytometry after staining with anti–human *CD4* and anti–mouse *Trbc* mAbs (left panels). Functional activity of transduced cells was tested by proliferation using a dye dilution assay after stimulation of transduced cells with the indicated peptides for 5 days. (**B**) Summary of specificity of expanded TCRs identified by scRNA-Seq in HC and T1D patients (newT1D and established T1D combined). Each rectangle represents an individual TCR. “Other” represents TCRs for which specificity was indeterminant or classified as bystander (i.e., iNKT-like TCR, Clone_197). iNKT, semi-invariant human natural killer T cell.

Key islet epitope(s) driving in vivo expansion of IAR T cells have not been identified. To demonstrate the specificity of expanded TCRs, we ascertained the specific cognate peptide(s) recognized by individual TCRs from the pools used to stimulate T cell activation. To accomplish this, we used recombinant lentiviral transduction methods to ectopically express TCR sequences in primary CD4^+^ T cells, followed by functional analysis of antigen specificity (26). We focused primarily on expanded TCRs because of our hypothesis that these are more likely key drivers of disease progression. In addition, we reasoned that a consensus sequence derived from multiple cells would be less susceptible to either single-cell sequencing or contig assembly errors. We selected a total of 47 TCRs in HC, newT1D, and T1D donors for specificity determination, primarily (45/47) from the most expanded TCRs. The rearranged *TRA* and *TRB* chain sequences were cloned into a lentiviral vector upstream of the murine *Tcra* and *Tcrb* constant regions. Primary human CD4^+^ T cells (usually DRB1*0401) were then transduced with recombinant lentiviruses, and cells that had or had not been transduced were identified by staining for the murine *Tcrb* constant region encoded by the recombinant TCR (Figure 1A) (44). The percentage of transduced cells averaged 78.5% + 8.84% (mean + SD).

Specificity of TCRs was determined by testing transduced T cells for proliferation in response to islet peptides (26). Cell division measured by CFSE dye dilution clearly distinguished specific versus nonspecific peptides (Figure 1A). In total, we identified islet peptides that triggered cell proliferation for 29/47 expanded TCRs tested (~62%). The specificities of tested TCRs are listed in Supplemental Table 3, and the results are presented graphically in summary (Figure 1B) and expanded forms (Supplemental Figure 2). We identified TCRs recognizing multiple islet antigens, indicating that expanded TCRs had multiple specificities. Most often TCRs recognized GAD65 peptides, followed by IGRP, then ZNT8 (Figure 1B), which may be due in part to more GAD65 peptides (18 or 20) in our 2 peptide pools than IGRP (3 or 5) or ZNT8 (6 or 7). Within GAD65 peptides, specific TCRs most often recognized the GAD65 113–132 amino acids peptide sequence (Supplemental Figure 3). Surprisingly, none of the TCRs we characterized recognized a well-studied epitope in preproinsulin (41). Thus, our multiplexed CD154 enrichment procedure was not equally effective at detecting all known islet antigen epitopes. There was no significant relationship between patient autoantibody profiles (Supplemental Table 1) and resulting TCR specificity, probably reflecting in part the low numbers of TCRs with specificity determined.

We found that measurable proliferation above background was dose-dependent upon peptide concentration, as shown for a subset of 23 expanded IAR TCRs in Supplemental Figure 2. Half maximal effective concentration (EC_50_) values are included in Supplemental Figure 3 and Supplemental Table 3. As a positive control, we used an influenza hemagglutinin (HA) peptide and its cognate HLA DRB1*0401-restricted TCR (45). Proliferation was maximal for all doses of HA peptide, indicating that the EC_50_ was less than the lowest concentration of peptide tested (EC_50_ < 0.01 μg/mL). Most IAR TCRs yielded dose-response curves shifted to the right (i.e., lower functional avidity) when compared with the HA reactive TCR, with EC_50_ values ranging from 0.01 to >10 g/mL (Supplemental Figure 3 and Supplemental Table 3). One of the 8 TCRs recognizing IGRP and all 4 TCRs recognizing ZNT8 were exceptions to this trend: these TCRs gave EC_50_ values less than the lowest concentration of peptide tested (EC_50 _< 0.01 μg/mL), showing greater avidity than other IAR TCRs. It may be important that these expanded, high-avidity TCRs were found only in single individuals. EC_50_ values were not clearly related to TCR transduction efficiency, suggesting that they were intrinsic to each TCR. In this small data set, EC_50_ values were not obviously related to disease status.

Not all TCRs from CD154^+^ cells triggered islet peptide-specific proliferation: 18/47 TCRs (~38%) did not proliferate in response to specific peptides (Figure 1B). TCRs that were not triggered by peptides in our tests may bind too weakly to induce proliferation in a recombinant system, which is a stringent readout. Alternatively, CD154 upregulation may be indirect for some TCRs. For example, we identified a group of TCRs sharing the invariant *TRA* junction, *TRAV*10-CVVSDRGSTLGRLYF -*TRAJ*18, paired with multiple *TRB* junctions. A BLAST comparison of this *TRA* chain against the nonredundant protein sequences database (http://blast.ncbi.nlm.nih.gov/blast.cgi) identified a perfect match with an iNKT receptor (46). iNKT receptors bind glycolipids presented by the MHC class I–related molecule, CD1d (47), and are not known to recognize islet peptides. To test specificity, we cloned and expressed one of the TCRs with the invariant iNKT-like *TRA* chain. We found that none of the individual islet peptides induced proliferation of TCR-transduced CD4^+^ T cells (Supplemental Figure 4), in contrast to the known iNKT ligand, α-galactosylceramide (Supplemental Figure 4). This suggests that CD154 upregulation was indirect for this iNKT-like TCR, perhaps because of bystander activation via IL-2 secreted by islet activated T cells (48). Thus, while most cells that upregulated CD154 were specific for the stimulating peptides, a smaller percentage of cells were of indeterminate specificity.

### Expanded public IAR TCRs are more prevalent in new-onset T1D.

To examine overall TCR diversity in our current data set, we compared Simpson’s diversity of TCR amino acid sequences across different experimental groups (Supplemental Figure 5). As diversity calculations, including Simpson’s diversity, are sensitive to the total number of TCRs, we downsampled to equivalent numbers of TCRs in different experimental groups before comparisons. In contrast to our previous study (26), differences in diversity metrics between HC and T1D patients were not significant in this expanded data set. Notably, however, TCRs from some newT1D and T1D patients had markedly lower diversity, reflecting greater TCR expansion, which was not seen in HC individuals. We obtained similar results using Shannon’s entropy as a diversity metric.

**Figure 2:**
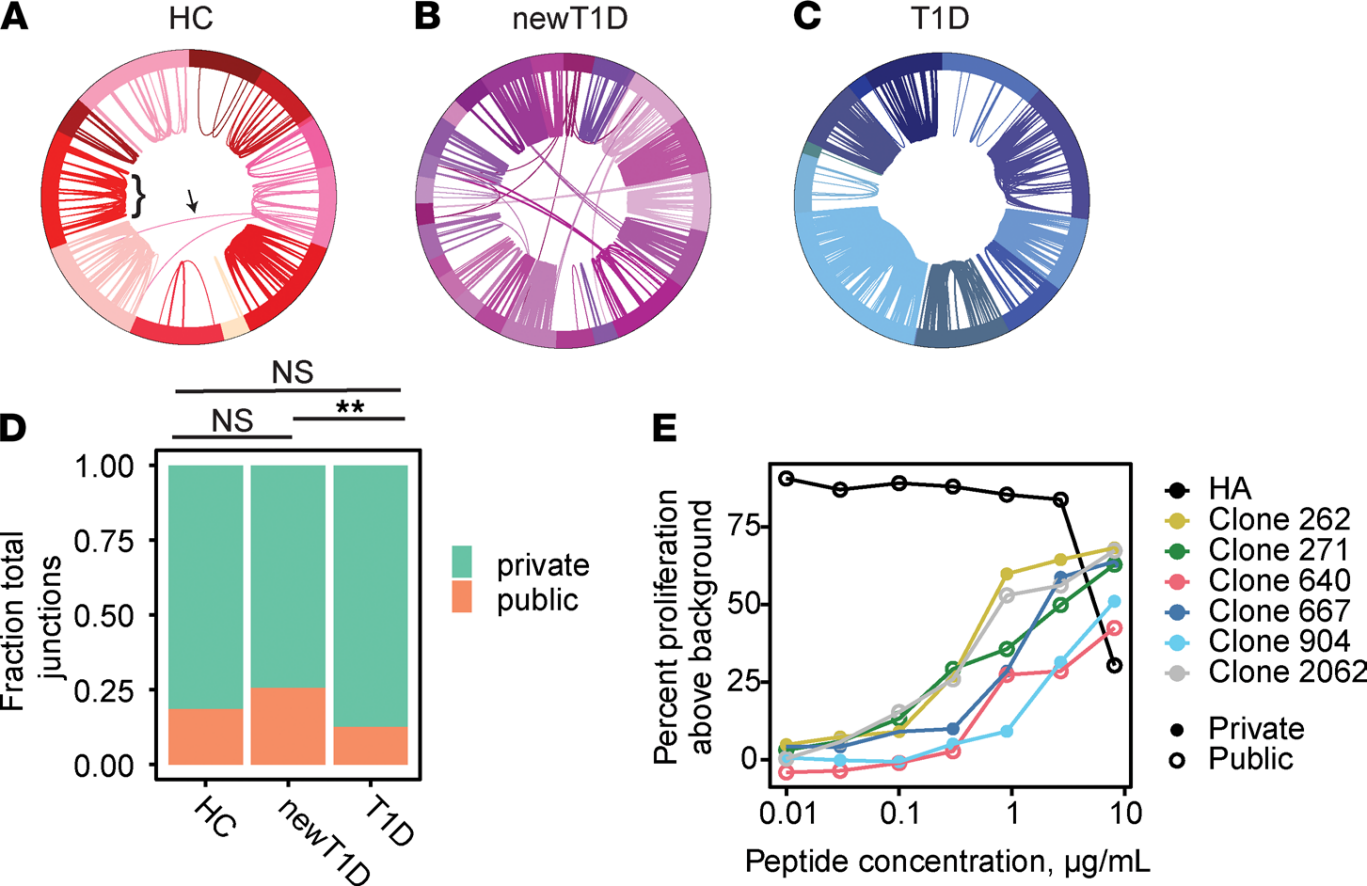
TCR diversity and clonotype sharing in IAR T cells. (**A**–**C**) Segments in the circos plots represent individual cells yielding *TRA* or *TRB* junctions listed in Supplemental Table 3 (*n* = 808, 1784, and 1481 filtered junctions for HC, newT1D, and T1D, respectively). Junction sharing (*TRA* or *TRB*) is indicated by arcs connecting different cells; arc thickness indicates number of junctions shared. Different donors are indicated by different colors in the outer ring. Arcs within donors represent expanded private junctions (bracket); arcs crossing between donors represent public junctions (arrow). (**A**) TCR sharing in IAR T cells from HC donors. (**B**) TCR sharing in individual IAR T cells from newT1D donors. (**C**) TCR sharing in individual IAR T cells from T1D donors. (**D**) Median numbers of public and private TCR chains vary by disease group. Frequencies of combined filtered public (*n* = 270) and private (*n* = 1130) junctions (Supplemental Table 3, public/private TCRs) were compared. Median junction numbers were tabulated in HC, newT1D, and T1D groups after iterative downsampling to equivalent numbers of expanded junctions (10,000 iterations, 183 junctions per subject group). Median numbers of public junctions were 34, 48, and 23 from HC, newT1D, and T1D, respectively. Significance of differences in public and private junctions by disease group was assessed using 2-by-2 contingency tables of numbers of public versus private chains by disease group, using Fisher’s exact test. **, FDR < 1 × 10^–^2; NS, not significant. (**E**) Public and private TCRs show similar functional avidity for GAD 113–132 versus a TCR recognizing an influenza HA peptide.

To assess TCR sharing, we compared rearranged junction amino acid sequences within and between HC, newT1D, and T1D donors. We detected extensive junction sharing within cells from the same individual in HC, newT1D, and T1D donors (Figure 2, A–C). In nearly all cases, junction identity extended to identity between V and J genes. Unlike our more limited earlier study (26), we also detected junction sharing between subjects, albeit at lower frequencies than sharing within patients. Overall, about 10% of total expanded TCR junctions were found in more than 1 donor (hereafter referred to as public), whereas about 90% of expanded TCR junctions were from a single donor (private). Classification of junctions as public or private is indicated in Supplemental Table 3. Public and private TCRs were subjected to additional filtering before use in comparisons below (Supplemental Methods).

Initial quantification of the levels of expanded public and private junctions by disease state revealed that the fraction of public junctions was elevated, and the fraction of private junctions was reduced, in newT1D, relative to HC and T1D, patients. It is possible that these results were biased because of unequal numbers of junctions, donors, and/or cells detected between disease groups. To insure against this potential bias, we repeated these comparisons following iterative random downsampling to equivalent numbers of junctions, donors, or cells between groups. Downsampling to equivalent numbers of junctions (Figure 2D) showed qualitatively similar results as the complete data set. Likewise, downsampling to equivalent numbers of donors or cells also gave similar results. We obtained similar results when using either total or unique junctions, again supporting a correlation with disease state. We also found that including or excluding naive cells from the designation of public/private junctions did not qualitatively alter our results. Another possible complication with these analyses was that HLA DRB1*03 genotype patients were only found among patients with newT1D. To separate the effect of HLA alleles on elevated public junctions, we excluded patients with HLA DRB1*03 genotypes from consideration. This analysis again yielded similar results, indicating that the elevation of public junctions in patients with newT1D was a function of disease state and not HLA type. Together, these results were robust and supported the conclusion that public junctions were elevated in newT1D, relative to HC and T1D, patients.

We examined functional avidity differences between public and private TCRs by comparing the dose-response curves for public and private TCRs recognizing the same GAD65 113–132 peptide (Figure 2E). Avidities of all public and private GAD65 113–132 specific TCRs were right shifted (lower avidity) relative to the HA peptide–TCR pair, with EC_50_ values ranging from 0.37 to 3.01 μg/mL. In this small sample set, there was not a significant difference in mean EC_50_ values for public versus private TCRs.

### Public TCRs are more germline like.

**Figure 3:**
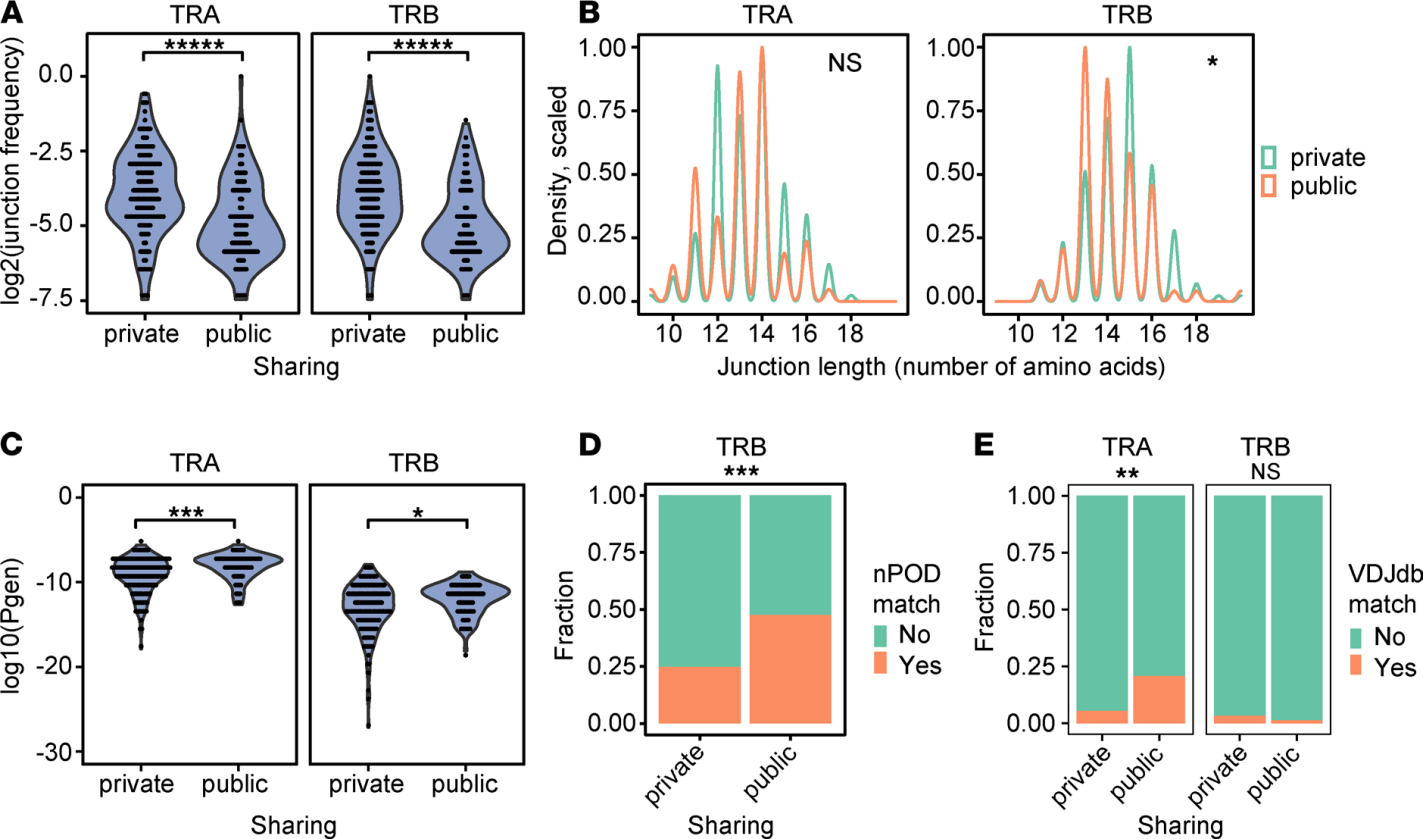
Public and private TCRs have different properties. (**A**) Frequency of private junctions was higher than public junctions. Frequencies of public (*n* = 270) and private (*n* = 1130) junctions (Supplemental Table 3, public/private TCRs) were compared. Public and private *TRA* or *TRB* junctions were combined, and the percentage of junctions in each class was calculated. Violin plots show the probability density of all data without summary statistics. The significance of differences between groups was determined using a 2-sided unpaired Wilcoxon’s signed rank test. *****, FDR < 1 × 10^–^5. (**B**) Private *TRB* junctions were longer than public junctions. Shown are the distributions of amino acid sequence lengths for all unique public and private *TRA* and *TRB* junctions (Supplemental Table 3, public/private TCRs) (*n* = 237 and 227 unique junctions for *TRA* and *TRB* chains, respectively). *, FDR < 0.05; NS, not significant. (**C**) Public *TRA* and *TRB* junctions were more germline like than private *TRA* chains. Shown are V(D)J generation probability values (*pgen*) for public (*n* = 270) and private (*n* = 1130) junctions (Supplemental Table 3, public/private TCRs), calculated using *IGoR* (50) software. Higher (less negative) *pgen* values indicate more germline-like V(D)J recombination compared to lower values. The significance of *pgen* differences between groups was determined using Wilcoxon’s signed rank tests. ***, FDR < 1 × 10^–3^; *, FDR < 0.05. (**D**) Public *TRB* chains show a higher fraction of *TRB* matches with sequences from pancreatic organ donors. Unique public and private *TRB* junctions (*n* = 78 and 149, respectively) (Supplemental Table 3, public/private TCRs) were tested for overlap with nPOD *TRB* junction sequences from spleen and lymph node (*n* = 2322 unique junctions). The significance of differences in frequencies of junction matches of public *TRB* chains with nPOD sequences was assessed by Fisher’s exact test. ***, *P* < 1 × 10^–3^. (**E**) Public *TRA* junctions show more perfect matches than private junctions with *VDJdb* junctions. Shown are the fractions of unique public and private *TRA* and *TRB* junctions (Supplemental Table 3, public/private TCRs) (*n* = 237 and 227 unique junctions for *TRA* and *TRB* chains, respectively) that overlap with *VDJdb* junctions (*n* = 47,069 unique junctions). 15/72 unique public *TRA* junctions had perfect matches with VDJdb sequences versus 9/165 public *TRA* junctions. **, FDR < 1 × 10^–2^.

Public and private T cell responses may be viewed as binary events or as extremes along a continuous spectrum of TCR sharing (14, 49). To probe the roles of public and private responses by IAR T cells in T1D, we compared properties of public and private TCRs. Within IAR T cells from all patients, the frequency of private junctions was higher than public junctions (Figure 3A). The amino acid sequence lengths of public and private junctions also differed. Public and private *TRA* junctions did not differ in median length (*n* = 13 amino acids), but public junctions were more variable in length than private *TRA* junctions (Figure 3B). Private *TRB* junctions were significantly longer than public junctions (median lengths of 15 versus 14 amino acids) (Figure 3B). These differences in length suggest that public junctions, especially *TRB* junctions, had fewer nontemplated nucleotides and, hence, were more germline like. To address the overall germline-like nature of public junctions, we examined the generation probability (*pgen*) of junction sequences during TCR recombination (50). Junction sequences with higher (less negative) *pgen* values have a higher probability of generation by V(D)J recombination. Conversely, sequences with lower (more negative) *pgen* values have a lower probability of generation. We calculated *pgen* values for public and private TCRs and compared them for both *TRA* and *TRB* junctions (Figure 3C). As expected, we found that *pgen* values were higher overall for *TRA* junctions than *TRB* junctions, in accordance with the reduced numbers of random nontemplated nucleotides in *TRA* junctions versus *TRB* junctions (14). We also found that *pgen* values for public IAR TCR junctions were higher than for private TCRs, especially *TRA* junctions (Figure 3C), indicating a higher probability of generation for public *TRA* junctions (i.e., more germline like). Higher *pgen* scores and shorter chain lengths seen in public IAR TCRs are interrelated properties of sequences common to many repertoires (14).

### Public TCR sequences are shared with TCRs from other sources.

To further characterize public and private TCRs from IAR T cells, we determined whether their junction amino acid sequences were represented in other TCR databases. One such database comprises TCR sequences from different T cell subsets isolated from spleen and pancreatic lymph nodes from autoantibody positive at risk (AAb^+^), T1D, and control donors collected through the Network for Pancreatic Organ Donors with Diabetes (nPOD) (27, 51). TCRs in this database are represented only by their *TRB* sequences. We found that public *TRB* junctions from IAR T cells from all donors had more perfect matches with nPOD-derived conventional T cell junctions, pooled from all sources, than did private junctions (Figure 3D). Among public *TRB* junctions, nearly half had perfect matches with nPOD-derived *TRB* junction sequences. In cases where the junction sequences matched, the V and J gene usage generally differed. These results demonstrated that junction sequences from peripheral IAR T cells were also found in immune cells located more proximal to the site of disease. Breaking down nPOD conventional T cell data by cell source (pancreatic lymph nodes and spleen) and disease state (AAb^+^, T1D, and control) gave qualitatively similar overlaps with IAR T cell *TRB* junctions.

*VDJdb* is another curated TCR database of TCR sequences with known antigen specificities, comprising both *TRA* and *TRB* junctions (52). When we tested for matches between *TRA* and *TRB* junctions from IAR T cells from all individuals and *VDJdb* amino acid sequences (Figure 3E), we found that about 21% of unique public *TRA* junctions (15/72 public *TRA* junctions tested) had perfect matches with *VDJdb* junctions, significantly more than private *TRA* junctions (Figure 3E). These matches were in junction regions and, generally, did not include V or J genes. Allowing single amino acid mismatches in the junction regions yielded qualitatively similar results, but the differences were not significant. In contrast, public and private *TRB* junctions had fewer matches than *TRA* junctions, whether perfect (Figure 3E) or single mismatches, and the number of matches did not differ significantly between public or private junctions. These findings could not be attributed to differences in numbers of *TRA* and *TRB* junctions compared, as there were ~1.8-fold more *TRB* than *TRA* junctions in *VDJdb* (52). Most *VDJdb* perfect matches with junctions from IAR T cell TCRs were junctions from TCRs targeting human cytomegalovirus (CMV) epitopes, identified by multiplex class I peptide–MHC multimer binding (53). Therefore, public *TRA* junctions from IAR T cells may share *TRA* junctions from TCRs with nonislet antigen specificities, including TCRs directed to CMV.

### Different chain pairings of public and private clonotypes.

**Figure 4:**
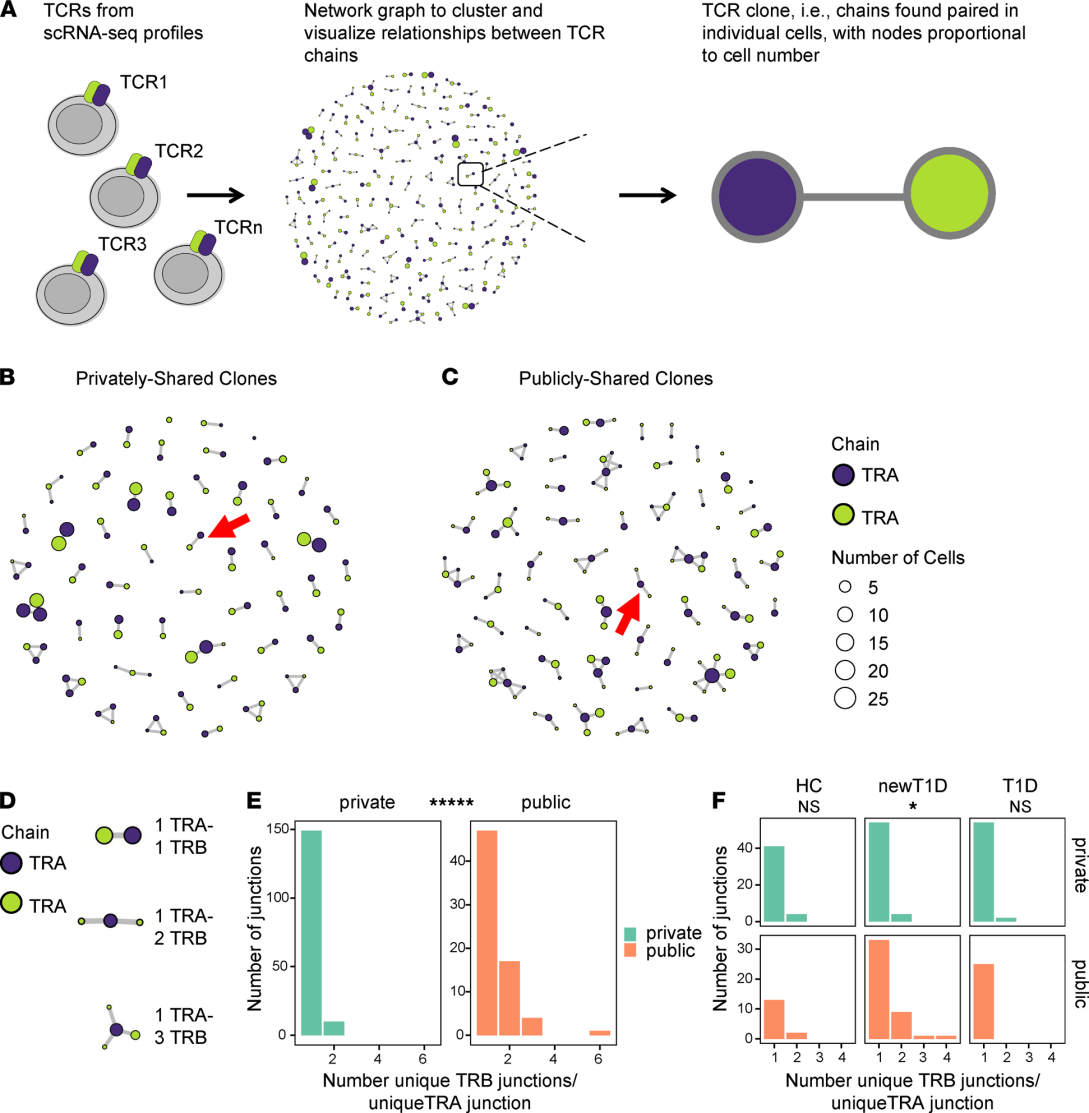
*tcrGraph* enables visualization of differing community structures of public and private clonotypes. (**A**) Clustering and network visualization of TCRs using *tcrGraph*. Each graph shows edges (lines) linking nodes (circles) of associated *TRA* (blue) and *TRB* (green) junctions. Node size is proportional to the number of cells containing a particular TCR chain, as indicated by the scale panel. (**B** and **C**) Network structures of public and private TCRs from all donors. For better visualization, private clones were randomly downsampled to equivalent numbers of clones as public TCRs (*n* = 55). Red arrows indicate major structures for each group (1 *TRA*-2 *TRB* and 1 *TRA*- 1 *TRB* for public and private clones, respectively). (**D**) Combinations of unique *TRB* chains associated with identical *TRA* chains identified by *tcrGraph*. (**E**) Numbers of *TRB* junctions paired with unique public and private *TRA* junctions were calculated (*n* = 72 and 165 unique public and private *TRA* junctions, respectively) (Supplemental Table 3, public/private TCRs). The significance of 1 versus multiple *TRB* junctions per *TRA* junction in unique public and private TCRs was assessed using a Fisher’s exact test. *****, FDR < 1 × 10^–5^. (**F**) Public *TRA* junctions were more associated with multiple *TRB* junctions in newT1D than in HC and T1D. As in **E** but broken down by disease group. *, FDR < 0.05; NS, not significant.

To probe more deeply into differences between private and public TCRs in IAR T cells, we took a network graph approach to analyze and visualize the landscape of TCR chain pairings. We developed a novel software package, *tcrGraph* (Supplemental Methods), that utilizes single-cell TCR chain sequence data to construct an undirected graph representing the TCR clonotype landscape for a given data set (Figure 4A). Each circle (vertex) in the graph represents a unique TCR chain, defined on the basis of either nucleotide or amino acid sequence. A line connecting 2 vertices (edge) indicates those junctions that were codetected in the same cell (i.e., “paired”) (Figure 4A). The size of each vertex is proportional to the number of unique cells where a chain was detected (clone counts). *tcrGraph* defines a TCR “clone” as a maximum number of chains that are paired with one another but not with other chains. We used *tcrGraph* to assign unique identifiers (cloneIDs) to each TCR clone (total TCRs) based on amino acid sequences (Supplemental Table 3).

We then used *tcrGraph* to generate network graphs for visualizing the landscape of TCR clones from public and private TCRs from IAR T cells (Figure 4, B and C). Using this approach, we found that the most prevalent clones with private TCRs were canonical *TRA*-*TRB* pairs (1 *TRA*-1 *TRB*) (Figure 4B and Supplemental Figure 6). In contrast, public TCRs unexpectedly showed prevalent clones comprising multiple *TRB* junctions in different cells associated with shared *TRA* junctions (1 *TRA*-2 *TRB*, etc.) (Figure 4C and Supplemental Figure 6). Tabulation of numbers of different junction pairings for public versus private TCRs for the entire data set confirmed that private TCRs were significantly enriched for *TRA*-*TRB* pairs (1 *TRA*-1 *TRB*) (Supplemental Figure 7). Chain pairings of 2 *TRA*-1 *TRB* have been implicated in development of autoimmunity (54), but frequencies of these pairings were not significantly different between public and private junctions. In contrast, public TCRs were enriched for more complicated structures involving different *TRB* junctions, found in different cells, but associated with shared *TRA* junctions (1 *TRA*-2 *TRB*, etc.) (Supplemental Figure 7).

To confirm and extend these findings from network graphing, we took a computational approach to determine different TCR chain pairings in public and private IAR TCRs from all patients in our study (Supplemental Table 3, public/private TCRs). We first defined different possible pairings of *TRB* and *TRA* junctions (Figure 4D) and then tabulated numbers of these pairings on a per cell basis (Figure 4E). This analysis showed that nearly all private *TRA* junctions were associated with a single *TRB* junction, whereas a significant number of public *TRA* junctions were associated with multiple *TRB* junctions (1 TRA-2 TRB, etc.). Breaking down these results by disease group showed that the fraction of *TRA* junctions associated with multiple *TRB* junctions (1 TRA-2 TRB, etc.) was greatest in newT1D patients (Figure 4F). A direct comparison of the fraction of multiple *TRB* junctions per *TRA* in newT1D versus T1D confirmed their difference (*P* = 0.00996, Fisher’s exact test). Notably, none of the public *TRA* junctions from patients with T1D were associated with multiple *TRB* junctions (Figure 4F). *TRA* chains were sometimes, but not always, shared across disease groups, with different *TRB* chains in different disease groups.

To ensure that the different chain pairing between public and private IAR TCRs was not biased by technical factors unrelated to disease state, we performed similar tabulations for the reverse pairings, i.e., numbers of *TRB* junctions associated with multiple *TRA* junctions in public and private TCRs. This test did not show that public *TRB* junctions shared more nonidentical *TRA* junctions (2 *TRA*-1 *TRB*, etc.) than private *TRB* junctions (Supplemental Figure 8). Thus, the differences in chain pairings in public IAR TCRs were more specific for multiple unique *TRB* junctions (1 *TRA*-2 *TRB*, etc.), consistent with biological specificity rather than randomly assorting technical factors.

### TRB junctions sharing a common TRA junction may have similar sequences and specificity.

**Figure 5:**
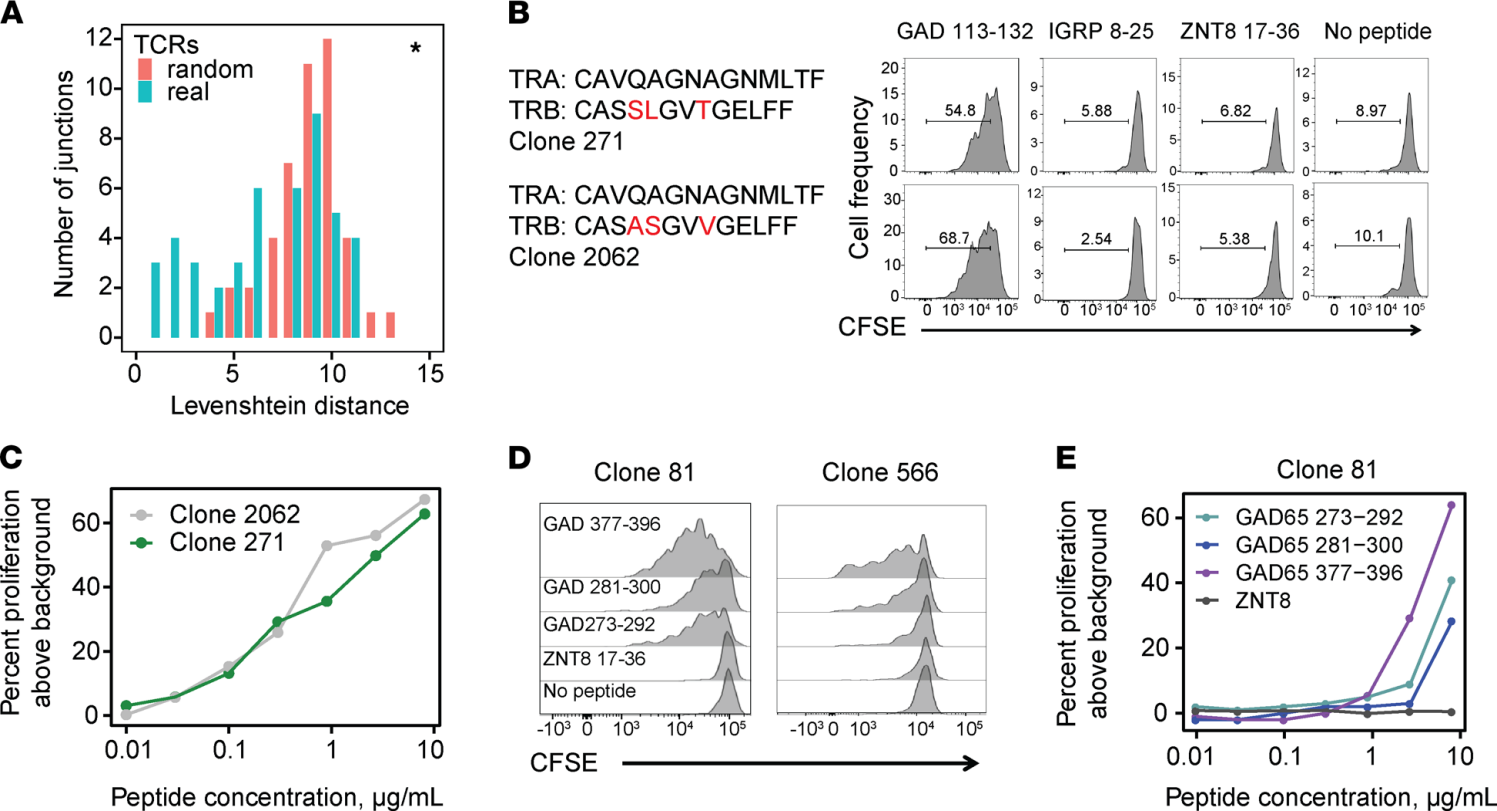
*TRB* junctions sharing a common public *TRA* junction differ in sequence but may have similar roles in binding. (**A**) *TRB* junctions that share *TRA* junctions show greater sequence identity than expected by chance. Numbers of *TRB* junctions paired with unique public and private *TRA* junctions were calculated from *n* = 72 and 165 public and private *TRA* junctions, respectively (Supplemental Table 3, public/private TCRs). Levenshtein distances were calculated for paired combinations of unique *TRB* junctions that pair with public *TRA* junctions (*n* = 31). For null sets, Levenshtein distances were calculated for *TRB* junctions in equal-sized, random sets of nonexpanded *TRB* junctions (*n* = 31 junctions) (Supplemental Table 3), and this was repeated *n* = 1000 times. Shown is a histogram representative of the median difference between real and random sets, as judged by *P* values from Kolmogorov-Smirnov tests. *, *P* value < 0.05. (**B**) TCR clones sharing public *TRA* chains with mismatched *TRB* junctions were functionally triggered by the same peptides. Recombinant TCR clones (Clone_271 and Clone_2062, Table 2) were transduced into primary CD4^+^ T cells, and proliferation was measured using a dye dilution assay following stimulation with the indicated peptides or a no-peptide control. Red font, mismatched residues. (**C**) Clone_271 and Clone_2062 TCR clones share similar dose-response curves for the GAD 113–132 peptide. (**D**) Cross-reactivity of related TCR clones, Clone_81 and Clone_566, for multiple GAD65 peptides. GAD 273–292 and GAD281–300 have overlapping sequences, but GAD 377–396 is distinct (Supplemental Table 2). (**E**) Dose-response curves showing cross-reactivity of Clone_81 for multiple GAD65 peptides and a nonreactive ZNT8 peptide.

To better understand pairing of multiple *TRB* junctions with an identical *TRA* chain in public IAR TCRs, we performed junction amino acid sequence comparisons. We identified the complete set of *TRB* junctions sharing *TRA* junctions (1 *TRA*-2 *TRB*, etc.) among all TCRs in our study and performed pairwise sequence comparisons. As a metric, we utilized Levenshtein distance, the minimum number of single amino acid residue changes (insertions, deletions, or substitutions) required to change one *TRB* sequence into the other. We then plotted the distribution of pairwise Levenshtein distances of unique *TRB* junctions associated with a single *TRA* chain compared with the distribution of distances between randomly selected *TRB* junctions from nonexpanded IAR TCRs (Supplemental Methods) (Figure 5A). While the distribution of pairwise distances between *TRA*-sharing *TRB* junctions was shifted significantly lower (more similarity) compared with the random sets (median *P* = 0.04, from 1000 different random sets, as compared by Kolmogorov-Smirnov tests), this was not true of all pairwise combinations. Thus, while some *TRA*-sharing *TRB* junctions from IAR T cells were similar in sequence, others were quite different.

To examine more closely sequence similarities between public *TRB* junctions associated with a single *TRA* chain, we performed sequence alignments for pairs with lowest Levenshtein distance (Figure 5A), rank-ordered by increasing Levenshtein distance (Table 2). This showed that *TRB* junctions with low Levenshtein distances were related sequences that shared perfectly matched *TRA* junctions and *TRB*V and *TRB*J genes.

TCRs with identical *TRA* chains but mismatched *TRB* junctions may or may not functionally recognize the same peptide(s). To resolve this question, we tested islet antigen specificity for public TCR clones with identical *TRA* chains and mismatched *TRB* junctions to determine if they recognized the same peptide (Table 2 and Figure 5B). Clone_271 and Clone_2062, which shared an identical *TRA* chain but had 3 amino acid mismatches in their *TRB* junctions (Table 2), both recognized GAD65 113–132 but not other peptides tested (Figure 5B). Both Clone_271 and Clone_2062 showed similar dose-response curves and EC_50_ values (0.8 and 0.4 μg/mL, respectively) (Figure 5C). In other experiments, we observed that cells transduced with Clone_81 and Clone_566 (Table 2 and Supplemental Table 3) showed similar proliferation responses to GAD65 377–396 peptide. These clones shared identical *TRA* chain sequences, *TRB*V and *TRB*J genes but had a single amino acid mismatch in their *TRB* junctions (Table 2). Thus, some of the IAR TCRs that share public *TRA* chains tolerated *TRB* junction mismatches without greatly disrupting peptide binding or subsequent T cell activation. While the number of examples of IAR TCRs tested is currently too small for firm conclusions, our results are reminiscent of the reported role of the *TRA* chain in the germline-governed recognition of a cancer epitope (55).

During our experiments determining TCR specificity, we noted that 3 TCRs (Clone_81, Clone_140, and Clone_566) showed specificity for multiple islet peptides (Supplemental Table 3). Multispecificity appeared more frequently in public (2/6 public clones tested, Supplemental Table 3) than in private TCRs (1/41 clones tested). Although these numbers were small, the difference in frequencies was weakly significant (*P* value = 0.0392, Fisher’s exact test). Examining the public multispecific TCRs in more detail, we noted that Clone_81 TCR was triggered by multiple peptides from GAD65. GAD 377–396 showed the strongest activity, while 2 additional peptides, GAD 273–292 and GAD 281–300, which overlapped each other but were noncontiguous with GAD 377–396, showed weaker activity (Figure 5D). This same pattern of multispecific activation was shown by the related but nonidentical clone, Clone_566 (Figure 5D). The pattern of nonspecificity was preserved in a dose-response comparison with Clone_81-transduced cells, with peptides GAD 273–292 and GAD 281–300 showing curves that were similar but shifted to the right (less activity) compared with GAD 377–396 (Figure 5E). GAD 273–292, GAD 281–300, and GAD 377–396 gave EC_50_ values of 6.23, 8.06, and 3.41 μg/mL, respectively. Importantly, peptide ZNT8 17–36 showed no activity at any dose tested (Figure 5E). Sequence comparisons between GAD 377–396 and GAD 273–292/GAD 281–300 showed no obvious primary sequence similarities. These results demonstrate that public IAR TCRs sharing *TRA* chains may be multispecific.

## Discussion

Using scRNA-Seq on a wider number of HCs and patients with T1D and broader duration of disease than we examined previously (26), we report several potentially novel findings. We show here that expanded IAR T cells recognize multiple islet antigen epitopes, and in contrast with inducible autoimmune models (18–21), do not show accumulation of immunodominant TCRs. We also found both public and private TCRs in T1D, but at different ratios according to disease duration. In accordance with our previous studies (26), IAR T cells in HCs and established T1D were predominantly private; public junctions were higher in newT1D samples, which we did not test previously. We showed previously that multiple private junctions were stable over consecutive visits, despite the fact that circulating T cell populations, especially more rare autoreactive populations, may be highly variable (26).

The frequency of public TCRs in IAR T cells was increased in newT1D relative to established T1D. This perhaps reflects dilution of specificities as epitope spreading occurs in established T1D (56–58). It is also possible that the reduction of public TCRs in established T1D reflects migration of islet antigen-specific T cells to the pancreas and adjacent peripheral nodes, or clone contraction due to antigen loss, during islet destruction. Expansion of public IAR TCRs near the time of clinical diagnosis suggests that even at their overall lower frequencies, they may play a role at disease onset.

Insulin-reactive T cell clones derived from islets of NOD mice that spontaneously developed T1D showed restricted use of *TRA* chains (23). The NOD clones were isolated early in disease (4–12 weeks) (23), when insulitis is evident but prior to development of diabetes (between 12 and 30 weeks) (59), perhaps corresponding to our observation of higher frequencies of public TCRs in newT1D versus T1D. Public TCRs targeting IGRP and utilizing restricted *TRA* chains were also seen in human T1D (29). In contrast, there are multiple examples of autoimmune disease models linked to autoreactive T cells with restricted *TRB* chains (18–21, 60). It is noteworthy that these previous reports of restricted *TRB* chains were all based on immunization-based models of autoimmune disease and exhibited immunodominant T cell clones that have been implicated as disease drivers (60). In contrast, our studies show a diverse spectrum of public IAR T cell clones linked to human T1D. That human T1D (29) and the spontaneous NOD mouse model (23) were associated with restricted *TRA* rather than restricted *TRB* chains may reflect different chain sharing with different disease-inducing mechanisms.

TCR repertoires represent a balance of specificity and cross-reactivity (61). Public TCRs from IAR T cells had more germline-like nucleic acid sequences and shorter *TRB* junction amino acid sequences than private TCR sequences. Lower sequence complexity would be predicted to decrease overall TCR specificity and increase cross-reactivity. Previous studies showed that *TRB* junction regions in polyclonal memory CD4^+^ T cell subsets were shorter in T1D than HC donors, which may increase the potential for self-recognition and heighten risk of autoimmune disease (62). Public IAR *TRA* junctions in our study also had more frequent matches to public TCR databases, particularly with TCRs that bind CMV peptide–MHC class I multimers. Our findings with CMV are reminiscent of a study showing T cell cross-reactivity of *GAD65*-reactive T cells with a peptide from human CMV (63). These previous findings were interpreted as evidence that molecular mimicry between islet autoantigens and the human pathogen, CMV, was a potential mechanism of autoreactivity in T1D (63). While our present findings are consistent with cross-reactivity, available evidence is inconclusive. Arguing against cross-reactivity are findings that matching was between class I– and class II–restricted TCRs and did not extend through the V genes. Cross-reactivity of islet peptide–MHC II and CMV *IE1*–MHC II complexes (61, 64) need not result from sequence similarity, as similarities in 3-dimensional spaces, or “hot spots” of structurally and chemically similar regions, may be more important for shared binding (64). Alternatively, our findings may reflect broader cross-reactivity of more germline-like and less complex public *TRA* chains for a variety of antigenic peptides. It will be important to design future studies to formally test on a global scale the cross-reactivity of public versus private islet reactive TCRs with peptide sequences from CMV and other sources. Also important will be focused studies on a more limited set of specificities, such as GAD 377–396, to better understand multispecific TCRs, such as Clone_81 and Clone_566.

Another potentially novel finding in our study was that public IAR TCRs were enriched for identical *TRA* chains paired with different *TRB* chains. The selection of identical *TRA* chains likely involves convergent recombination (17) and suggests that *TRA* chains may play a dominant role in antigen binding. TCRs with dominant *TRA* chains are potentially limited in antigen specificity and affinity. One limitation is because *TRA* chains are inherently shorter and less diverse than *TRB* chains. In addition, because *TRA* chain rearrangement occurs after expansion of cells with rearranged *TRB* chains, each convergently rearranged *TRA* chain will encounter a limited number of rearranged *TRB* chains. The limited diversity of TCRs bearing identical *TRA* chains imposes an additional constraint on TCR specificity and affinity. We suggest that these constraints on TCR specificity and affinity increase the potential for self-recognition by germline-like and less complex public *TRA* chains and may confer increased risk of autoimmune disease. Future studies on the relevance of public *TRA* chains to TCR repertoires targeting infectious agents and other antigens may help clarify how unique these chains are to autoreactive T cells.

A limitation of our study is the relatively limited size. While more highly powered than our previous studies (26), the current study remains underpowered to elucidate effects of confounders such as age, sex, and ethnicity on our results. Additionally, undersampling of either patients and/or cells would result in underestimating the frequency of public versus public IAR TCRs, respectively, and concomitant overestimating of private TCRs. The degree of sharing of IAR TCRs detected depends both on sequencing depth and cohort size (14). The limitation on numbers of cells sequenced per subject directly reflects the low frequency of IAR T cells in peripheral blood. Mitigating this restriction will likely require a more enriched source of cells, possibly the pancreas or draining lymph nodes from cadaveric specimens (27). In the present study, limitations on numbers of cells sequenced may have contributed to our inability to detect a shared GAD65-specific TCR (65) that has been found in patients with T1D (27) or preproinsulin-reactive TCRs. It will be important to confirm our results using an independent validation data set.

The use of TCRs for biomarkers and therapeutic targets in T1D is an area of active research (28). Previous studies showed a lack of overlap between TCRs from bulk CD4^+^ T cells in tissues and circulation, suggesting that TCRs from peripheral blood may lack utility as biomarkers and may not play a causal role in disease (27). However, there are several aspects of our present study that were not addressed previously to our knowledge. First is our focus on IAR T cells, which are rare in peripheral blood but more likely to play a causal role in disease than the bulk T cells examined previously. Another feature is our demonstration of TCR sharing between individuals (i.e., public chains). These public chains are more practical for translational applications than private specificities, which are unique to each individual. While our results do not support the presence of single immunodominant islet reactive TCRs, we did find shared *TRA* public chains in a sizable fraction of newT1D/T1D patients (up to ~5/27 total donors, or ~18%). The figure is likely to be an underestimate, as our methods detected relatively low numbers of cells. In addition, the use of combinations of restricted public clones may allow coverage of an even larger and more useful fraction of newT1D/T1D patients. However, we recognize that unless most or all drivers of disease are among the public clones, targeting cells expressing these public clones is not likely to modify disease. Another caveat to translational uses of shared *TRA* public chains from IAR T cells is potential cross-reactivity of these TCRs with CMV or other microbes. Using such cross-reactive public TCRs as targets for T cell depletion might introduce holes in the host antimicrobial repertoire, although it is not known how consequential any such holes would be for the overall antimicrobial repertoire. Finally, the predominance of public *TRA* chains in IAR T cells indicates the need for additional translational studies to evaluate *TRA* chains as well as *TRB* chains. This is especially true because *TRB* chains have been the focus of most studies investigating TCRs as biomarkers and therapeutic targets (28). Thus, we suggest that more complete data on both *TRA* and *TRB* chain usage and pairing in IAR T cells are needed to make firmer conclusions regarding correlation or causality of TCR chains with disease, and/or their relevance for T1D therapeutics.

## Methods

### Experimental methods.

Methods are available as Supplemental Methods.

### Repository information.

Code for *tcrGraph* is available at https://github.com/BenaroyaResearch/tcrGraph (commit ID: 4508d72c46e3c830230a49525fcc8aea893666e1). Code and data for generating the figures are provided at https://github.com/BenaroyaResearch/Shared-germline-like-TCR-alpha-chains-in-type-1-diabetes (commit ID: fb926c7aaf8f72203eb72f4bb0cb4c9481e233be). Profiles yielding TCRs were deposited in the National Center for Biotechnology Information Gene Expression Omnibus repository (accession number GSE182870; https://www.ncbi.nlm.nih.gov/geo/).

### Study approval.

Protocols for these studies were approved by the Institutional Review Board of Benaroya Research Institute (IRB7109-332). Protocols for the T1DAL clinical trial were approved under the auspices of NCT00965458, as described previously (38).

## Author contributions

EB, JC, and FBW performed and KC directed laboratory experiments; VHG directed RNA-Seq profiling; MGR performed pipeline analysis of the RNA-Seq data and developed the *tcrGraph* software; PSL directed and PSL, EB, HAD, KJF, and AKH analyzed RNA-Seq data; CO performed EC_50_ analysis; WWK, CS, CJG, GTN, ES, KM, HRS, and TMB provided materials; PSL, KC, FBW, EB, HAD, KJF, and HKU interpreted data; PSL and KC conceived the experiments; and PSL and KC wrote the manuscript. All authors made contributions to the final manuscript.

## Supplementary Material

Supplemental data

Supplemental table 1

Supplemental table 2

Supplemental table 3

Supplemental table 4

## Figures and Tables

**Table 1:**
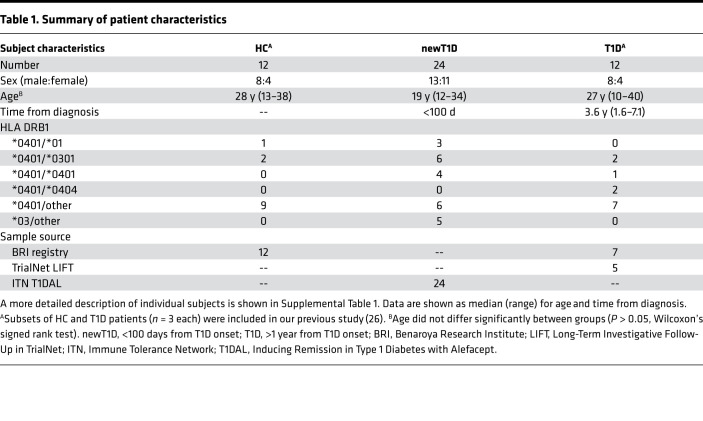
Summary of patient characteristics

**Table 2:**
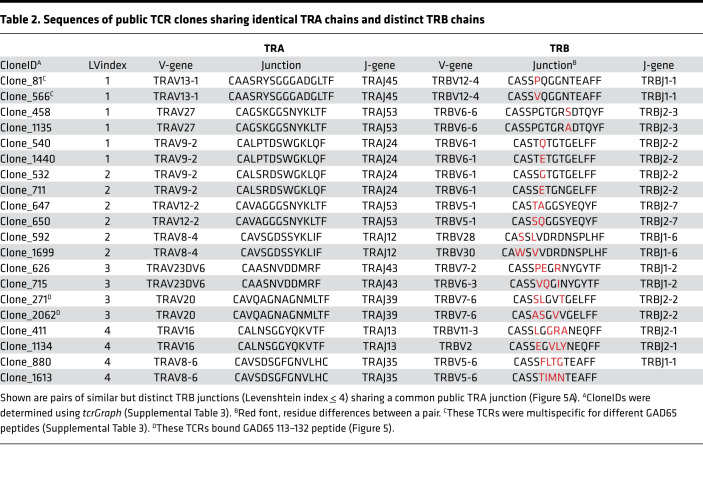
Sequences of public TCR clones sharing identical TRA chains and distinct TRB chains

## Version 1 11/22/2021

Electronic publication
