# Corrigendum to: Autoreactive T cell receptors with shared germline-like α chains in type 1 diabetes

**DOI:** 10.1172/jci.insight.207149

**Published:** 2026-04-22

**Authors:** Peter S. Linsley, Fariba Barahmand-pour-Whitman, Elisa Balmas, Hannah A. DeBerg, Kaitlin J. Flynn, Alex K. Hu, Mario G. Rosasco, Janice Chen, Colin O’Rourke, Elisavet Serti, Vivian H. Gersuk, Keshav Motwani, Howard R. Seay, Todd M. Brusko, William W. Kwok, Cate Speake, Carla J. Greenbaum, Gerald T. Nepom, Karen Cerosaletti

Original citation: *JCI Insight*. 2021;6(22):e151349. https://doi.org/10.1172/jci.insight.151349

Citation for this corrigendum: *JCI Insight*. 2026;11(8):e207149. https://doi.org/10.1172/jci.insight.207149

After publication, the authors became aware of errors in Table 2. The correct table is provided below and has been updated in the HTML and PDF version of the article.

The authors regret the error.

## Figures and Tables

**Table 2:**
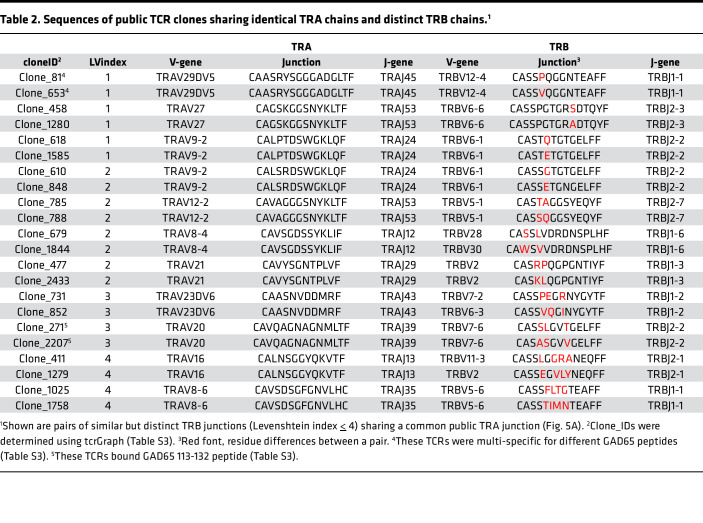
Sequences of public TCR clones sharing identical TRA chains and distinct TRB chains.^1^

## Version 1 04/22/2026

Electronic publication

