# Supplementary material for: Autoreactive T cell receptors with shared germline-like α chains in type 1 diabetes
Source: JCI Insight. 2021 Nov 22;6(22):e151349. doi: 10.1172/jci.insight.151349 (PMC8663791; doi:10.1172/jci.insight.151349)

Title: Autoreactive T cell receptors with shared germline-like alpha chains in type 1 diabetes

Supplemental Methods

Human subjects. Whole blood and banked PBMC samples were obtained from HC and established T1D subjects with informed consent (Table I). HC were matched for age and sex to T1D patients and had no personal or family history of T1D. Some of the T1D and HC subjects (N = 3 each T1D and HC) were described in an earlier report (1). HC, T1D and most newT1D subjects had high-risk *DRB1*0401* HLA class II alleles. Patient characteristics are summarized in Table I and in more detail in Table SI.

Isolation of IAR T cells. Peripheral blood was drawn by venous puncture using heparin as anti-coagulant. Peripheral blood mononuclear cells (PBMC) were isolated by Ficoll-Hypaque centrifugation and were used fresh or were banked. Information on whether frozen or fresh cells were used with each donor is provided in Table S1. For HC (N = 14 total samples), cells from 6 donors were used fresh, and cells from 8 frozen; for newT1D (N = 24 total samples), all were used frozen; and for T1D (N = 21 total samples), cells from 11 donors were used fresh, and cells from 10 frozen. Expanded IAR T cells from frozen and fresh samples did not differ in fractions of public versus private TCRs.

With a few exceptions, procedures for isolation of IAR T cells antigen (Figure S1) were essentially as described previously (1). Briefly, fresh or frozen PBMC were cultured in either 48 well or 6 well plates at 10e6 cells/ml in RPM1 1640 supplemented with 10% commercial human serum (Gemini Bio Products, West Sacramento, CA), penicillin/streptomycin (100 U/ml, 100 µg/ml), sodium pyruvate (1 mM), and L-glutamine (2 mM) and anti-CD40 antibody (Miltenyi Biotec, San Diego CA) at 37°C. Cells from HC and T1D subjects were stimulated for 14h with a pool of 28 islet antigen peptides (HLA DRB1*0401 restricted) at 1.7 µg/ml. Cells from newT1D subjects were cultured with a pool of 35 islet peptides (*HLA-DRB1*0401/0301/DQ8* restricted pool) at 1.4 µg/ml to accommodate the minority of newT1D subjects who had *DRB1*0301* alleles (Tables 1 and S2). As controls, cells were cultured in the presence of an equal volume of DMSO (negative control) or a mix of viral peptides (positive control) for 14h. Viral peptides included: influenza *MP* peptides amino acids 57–76 and amino acids 97–116

(KGILGFVFTLTVPSERGLQR, VKLYRKLKREITFHGAKEIS); and overlapping peptides from the CMV *pp65* and *AdV5* hexon gene products (Miltenyi Biotec). PBMC were then stained with anti-CD154-phycoerythrin (PE) labeled antibody (Miltenyi Biotec) followed by anti-PE coupled magnetic beads (Miltenyi Biotec), and CD154+ cells were enriched by magnetic bead separation. Enriched cells were surface stained with the antibodies indicated in Table S4 and CD4+CD154+CD69+ cells (naïve and memory cells for newT1D samples, memory cells only for HC and T1D samples) were single cell sorted using a BD FACSAria flow cytometer with the gating scheme shown in Figure S1B. For each sample, the CD154+CD69+ gate in islet stimulated cells was set based on the DMSO treated control. One variation from our previous protocol was that we sorted cells directly into 96 well plates (Figure S1A) instead of microfluidic chips for most of these experiments. The stimulation conditions utilized did not result in detectable cell division during the culture period (1). Since regulatory T cells do not strongly up-regulate CD154 under these experimental conditions (2), the isolated cells (Figure S1B) were predominantly effector T cells.

Lentiviral constructs and viral production. Oligonucleotides (Genscript, Piscataway, NJ) encoding codon-optimized expanded rearranged *TRAV* and *TRBV* sequences were cloned (1) into the lentiviral plasmid, pRLL-MND-GFP (3), which was kindly provided by Dr. David Rawlings (Addgene plasmid #36247). In some cases, *TRA* and *TRB* sequences were first cloned into the modified 'TCR flex' pMP71 retroviral backbone (kindly provided by Dr. Ton Schumacher) upstream of the murine *Trac* and *Trbc* genes (4). The cloned *TRAV-TRBV* open reading frame in pMP71 was transferred as a Not1 and SpeI restriction fragment in place of the green fluorescent protein (GFP) sequence in pRLL-MND-GFP (3). For lentivirus production, 293T (ATCC CRL -3216) packaging cells were plated into 10-cm dishes and grown to ~95% confluency. Cells were transfected with packaging, envelope, and transfer plasmids using Polyethylenimine (PEI). Viral-containing supernatants were collected at 48 hrs. The virus was precipitated with PEG-it (System Biosciences) and concentrated 100x. Viral titers were determined using Lenti-X GoStix Plus (Takara).

Transduction of human CD4$^+$ T cells. CD4+ T cells (1e6) were isolated from human PBMC using magnetic beads (Miltenyi Biotech, San Diego, CA) and stimulated for 48 hrs in 24 well plates with ImmunoCult™ human CD3/CD28 T cell activator in ImmunoCult™-XF T Cell Expansion Medium (STEMCELL Technologies, Vancouver, BC, Canada) supplemented with 100 IU ml−1 rhIL-2 and 5µg/ml of rhIL-7 and rhIL-15 (growth medium). For lentiviral transduction, 5-20 microliters of 100x virus added to activated CD4$^+$ T cells (0.2e6 in 150 microliters T cell expansion medium supplemented with 10µg/ml protamine sulfate). Cells were spin inoculated by centrifugation of culture plates for 30 minutes at 500g, then rested for 18 hrs at 37°C in fresh growth medium. After 3 days, transduction efficiency was determined by staining with a monoclonal antibody (mAb) targeting the murine *Trbc* chain (H57-597, allophycocyanin (APC)-labeled, BD Biosciences, San Jose, CA).

Antigen specific proliferation assays. Peptide-induced proliferation was detected by CFSE dye dilution for transduced CD4$^+$ T cells (1, 5). KRN 7000 (Avanti Polar Lipids, Inc., Alabama/USA) was used to trigger iNKT cell-like proliferation. Since autologous antigen presenting cells were not always available for these experiments, we instead used banked PBMC (irradiated) from an unrelated donor positive for *DRB1*0401* (or *DRB1*0301* and *DQ8*) class II molecules. In some cases, we also used the Priess lymphoblastoid cell line (DRB1*0401, *0401) as APC. Although these cells expressed DRB1*0401, *0301, and/or DQ8, they likely had other HLA mismatches with patient cells (MHC class I, etc.). To control for the confounding effects of alloantigen responses driven by HLA mismatched APC, we set gates on cells cultured with no peptide. We then compared responses of transduced T cells, which exhibit both islet peptide- and alloantigen-driven responses, with non-transduced cells, which exhibit only alloantigen responses. Since ~70% of cells are transduced and ~30% of cells in the cultures are not transduced, we also compared proliferation of both cell subsets in the same cultures by gating on anti-murine Trbc stained cells (reference (1); see also Figure. S4). We typically observed minimal proliferation in either transduced or un-transduced T cells in the absence of peptide, indicating that alloreactivity was not a major concern under these conditions. Although the numbers of public TCRs for which we determined specificity was small, we noted no

significant differences with private TCRs in no peptide control values. Indicator CD4+ T cells were labeled with 5(6)-Carboxyfluorescein diacetate N-succinimidyl ester (CFSE). PBMCs isolated from a healthy HLADRB1 0401/0301 individual were used as antigen presenting cells and were loaded with antigenic peptides, which unless otherwise stated, were added to a final concentration of 5 µg/ml. CFSE-labeled CD4+ T cells (1e4) were mixed with 4e4 irradiated antigen presenting cells and then cultured for up to 5 days at 37°C in a 5% CO2 incubator. Finally, cells were stained with anti- human CD4+ (Clone RPA-T4, phycoerythrin-labeled, BioLegend, San Diego CA), and the murine Trbc chain (H57-597, allophycocyanin (APC)-labeled, BD Biosciences, San Jose, CA) to identify transduced cells. (1). CFSE intensities in different cell populations were then measured by flow cytometry and quantified by gating on the murine TCR^+ or TCR^- populations in the $CD4^+$ parent population.

EC50 determinations. EC50 concentrations were determined using Bayesian curve fitting. Growth was assumed to follow the logistic curve defined as

$$y = \frac{2\mathrm{H}}{1 + \mathrm{e}^{\frac{2\mathrm{D}(\mathrm{M}-\mathrm{x})}{H}}}$$

where M is the EC50 value, H is the height of the curve at EC50, and D is the slope of the curve at EC50. We fit Bayesian hierarchical growth curve models to the data using Stan, a C++ library for Bayesian inference (6), in R using the *cmdstanr* interface (7). The posterior distributions of the parameters were simulated using MCMC sampling 4 chains each with 7,000 post-warmup iterations. Point estimates for EC50 values use the median of the posterior distributions.

Cell capture, scRNA-seq and TCR clonotype identification. The frequencies of IAR $CD4^+$ memory T cells are low, especially in frozen PBMCs (a median of ~49 cells per sort (72 sorts) were recovered in our experiments). A major advance from our previous work (1) was development of the capability to sort islet- antigen reactive T cells directly into 96 well plates, which increased the cell capture yield, and resulted in better quality libraries. In agreement with our previous studies (1), we did not observe consistent differences in the numbers of cells captured nor the number of high quality libraries recovered between HC, newT1D and T1D subjects. After capture, cells were processed into libraries which were sequenced on a

HiSeq2500 sequencer (Illumina, San Diego, CA) using primarily 58 bp single-read dual-indexed reads (1). Methods for RNA-seq pipeline analysis and TCR clonotype identification were essentially as described previously (1, 8), except that we used the *MiXCR* (9) software package to identify productive TCR chain rearrangements. Here we employ the IMGT (10) convention of using junction to refer to the amino acid sequence starting with the 2nd cysteine residue encoded in the V gene and ending with the phenylalanine or typtophan residue in the J gene. Quality control filtering metrics included: the presence of an in-frame rearranged TCR chain, shown previously to correlate well with more extensive quality metrics (1); TCR junction length <30 amino acids; *TRAV* or *TRBV,* but not *TRDV* or *TRGV,* gene usage; and two or fewer *TRA* chains and two or fewer *TRB* chains. These TCRs are listed in Table S3 (total TCRs). For analyses, we additionally filtered out iNKT-like TCRs and TCRs from non-memory cells (CD45RA index marker positive). TCRs retained after quality control and filtering are denoted in Table S3 (filtered TCRs). For some analyses, public and private TCRs were retained after additional filtering for type of junction sharing, also denoted in Table S3 (public/private TCRs).

tcrGraph. We created the *tcrGraph* package in the R statistical computing language to implement a *tcrGraph* S3 class and provide utility functions for graph construction, data visualization, clone counting, and conversion to graph formats for other popular analysis packages such as *igraph*. Code is provided at https://github.com/BenaroyaResearch/tcrGraph.

Sequence comparisons. Pairwise Levenshtein distances between peptide sequences were calculated using the *stringdist* software package. For calculating the significance of a test set of Levenshtein distances between *TRB* junctions, we devised a permutation testing procedure. We first took all unique *TRB* junctions in our data set (Table S3) and removed expanded public and private junctions. We next selected a random subset of these *TRB* junctions of equal size to the original test set, then calculated pairwise Levenshtein distances between the random junctions. We then calculated differences in the distributions of Levenshtein distances for the test set versus the random set using Kolmogorov–Smirnov (KS test) tests. We repeated this process 1,000 times to generate a distribution of KS test *p* -values.

Finally, we determined overall significance as the median KS test p-value for differences between the test set versus random sets.

Statistics. Statistical tests were performed using the R programming language and software environment. For continuous, normally distributed variables, we utilized t-tests; for non-normally distributed variables, Wilcoxon signed rank tests; and for categorical variables, Fisher's exact test. Unless otherwise noted, two-sided tests were performed. Where appropriate, multiple testing corrections were made (11). The term "significant" is reserved for p-values or false-discovery rates (FDR) of <0.05. The specific test used to derive each p-value is listed in the Figure legend. Hypergeometric p-values were calculated using the *phyper* function in R. Simpsons diversity was calculated as described (12). Where indicated, asterisks were used to indicate the significance level of p-values (or FDRs): *****, <1e-5; ****, <1e-4; ***, <1e-3; **, <1e-2; and *, <5e-2.

References

1. Cerosaletti K et al. Single-Cell RNA Sequencing Reveals Expanded Clones of Islet Antigen-Reactive CD4(+) T Cells in Peripheral Blood of Subjects with Type 1 Diabetes. *J. Immunol. Baltim. Md 1950* [published online ahead of print: May 31, 2017]; doi:10.4049/jimmunol.1700172

2. Schoenbrunn A et al. A converse 4-1BB and CD40 ligand expression pattern delineates activated regulatory T cells (Treg) and conventional T cells enabling direct isolation of alloantigen-reactive natural Foxp3+ Treg. *J. Immunol. Baltim. Md 1950* 2012;189(12):5985–5994.

3. Astrakhan A et al. Ubiquitous high-level gene expression in hematopoietic lineages provides effective lentiviral gene therapy of murine Wiskott-Aldrich syndrome. *Blood* 2012;119(19):4395–4407.

4. Linnemann C et al. High-throughput identification of antigen-specific TCRs by TCR gene capture. *Nat. Med.* 2013;19(11):1534–1541.

5. Lyons AB, Parish CR. Determination of lymphocyte division by flow cytometry. *J. Immunol. Methods* 1994;171(1):131–137.

6. Stan Development Team. *Stan Modeling Language Users Guide and Reference Manual [Internet]*

7. Gabry, J, Cesnovar, R. *cmdstanr: R Interface to "CmdStan" [Internet]*. 2020:

8. Long, S.A., et al. Partial exhaustion of CD8 T cells and clinical response to teplizumab in new-onset type 1 diabetes. *Sci. Immunol.* 2016;1(eaai7793):1–9.

9. Bolotin DA et al. MiXCR: software for comprehensive adaptive immunity profiling. *Nat. Methods* 2015;12(5):380–381.

10. Alamyar E, Duroux P, Lefranc M-P, Giudicelli V. IMGT(®) tools for the nucleotide analysis of immunoglobulin (IG) and T cell receptor (TR) V-(D)-J repertoires, polymorphisms, and IG mutations: IMGT/V-QUEST and IMGT/HighV-QUEST for NGS. *Methods Mol. Biol. Clifton NJ* 2012;882:569–604.

11. Hochberg Y, Benjamini Y. More powerful procedures for multiple significance testing. *Stat. Med.* 1990;9(7):811–818.

12. Jost L. Entropy and diversity. *Oikos* 2006;113(2):363–375.

Title: Autoreactive T cell receptors with shared germline-like alpha chains in type 1 diabetes

Supplemental Tables

Table S1. Subject characteristics.

Table S2. Islet antigen peptides used for T cell stimulation.

Table S3. TCR sequences from single cell RNA-seq experiments

Table S4. Antibodies used for flow cytometric isolation of IAR T cells

Supplemental Figures

Figure S1. Experimental schema. A) Isolation of islet antigen reactive CD4 T cells using a CD154 activation and enrichment assay. PBMC were stimulated with a pool of immunodominant islet peptides (Table S2), viral peptides or vehicle control in the presence of anti-CD40 antibody for 14h. Cells were stained with an anti-CD154-PE couple antibody followed by anti-PE coupled magnetic beads. CD154+ cells were enriched on a magnetic column, eluted, surface stained, and analyzed by flow cytometry. Islet peptide reactive CD4 T cells were single cell sorted into 96 well plates for subsequent scRNA-seq. **B)** Flow cytometry gating strategy for antigen reactive CD4 T cells, defined as CD154+CD69+. The gate for CD154+CD69+ islet reactive CD4 T cells was set based on the DMSO negative control for the same donor as shown in (A).

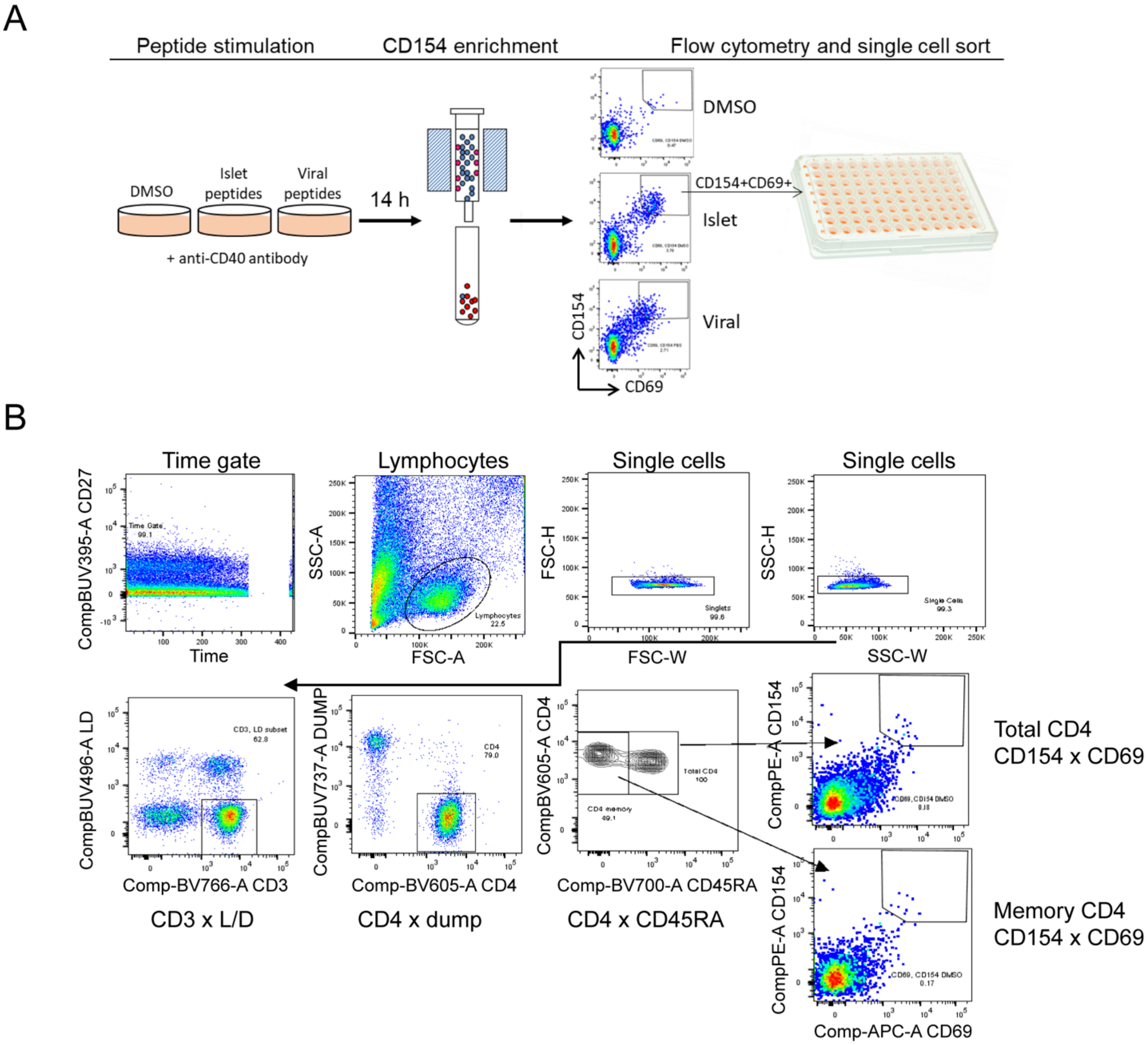

Figure S2. Epitope specificities of individual TCRs identified by scRNA-seq in HC and T1D subjects. Individual TCRs were re-expressed by lentiviral transduction, and islet peptide specificity was determined by proliferation assays. Shown are islet epitopes recognized by TCRs isolated from combined newT1D and T1D (red), or HC subjects (cyan). Each TCR with a given specificity is represented by a box. Other, TCRs that did not trigger proliferation in response to a unique islet peptide epitope, or were classified as bystander (i.e., iNKT-like TCR, Clone_197).

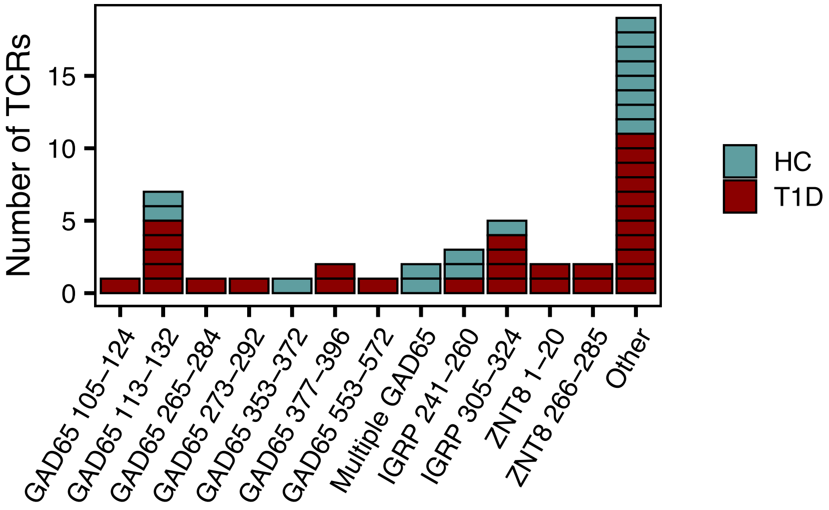

Figure S3. Dose response curves for selected islet antigen reactive TCRs. Shown are results of dye dilution assays to determine the EC50s of expanded islet reactive TCRs isolated from HC, newT1D and T1D subjects. Lentiviral-transduced TCR CD4 T cells were labeled with CFSE and stimulated with varying concentrations of the indicated islet antigen peptides for 5 days. Dye dilution was analyzed by flow cytometry and is reported as the percent of proliferated cells above the no peptide control for the same TCR. Each graph represents an individual islet reactive TCR (n=23), with an HA reactive TCR as a positive control (lower right). EC50 values are reported as μg/ml.

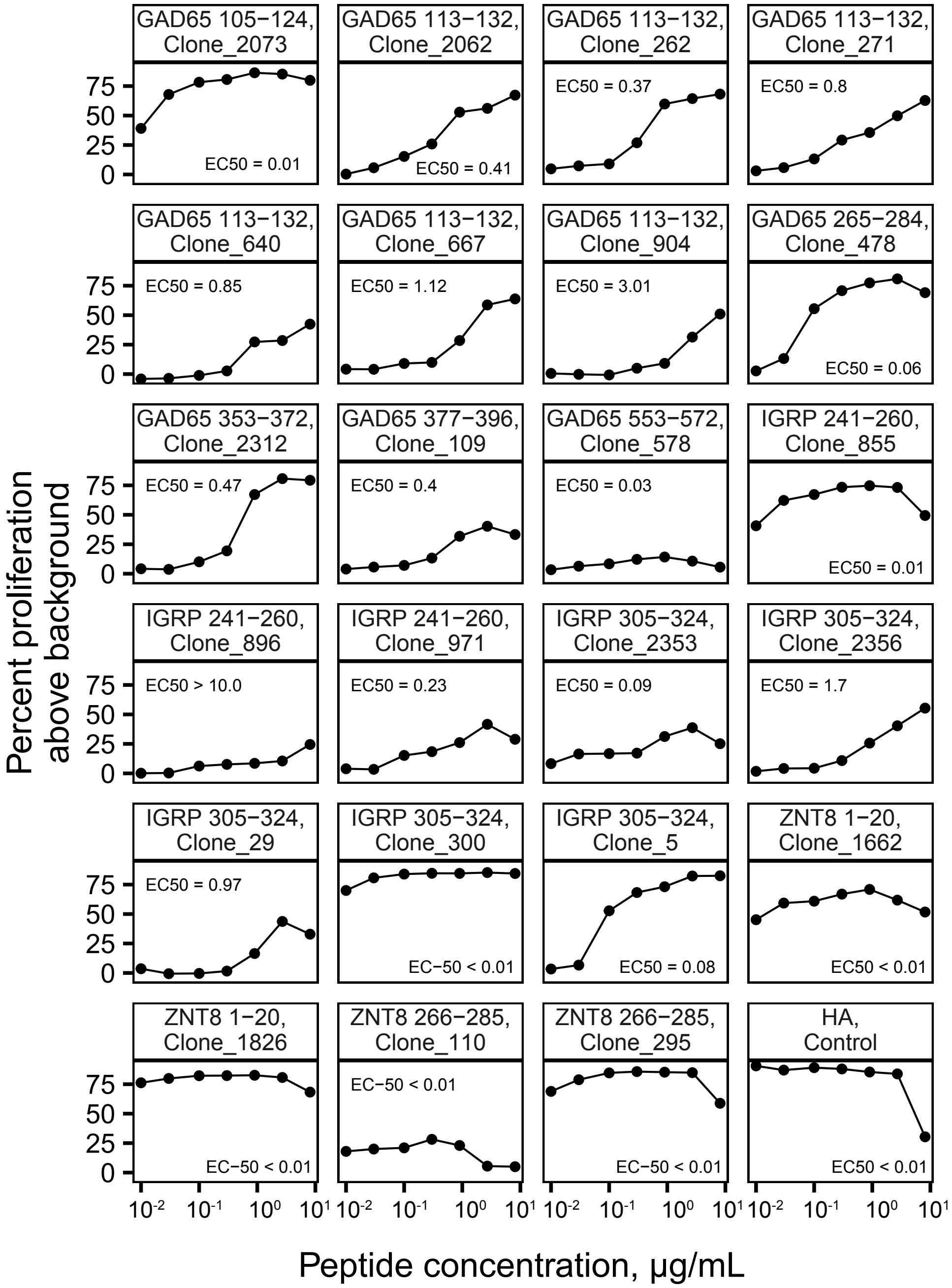

Figure S4. Specificity of an iNKT-like TCR for galactosylceramide, not islet peptides. Shown are results of dye dilution assays of CD4 T cells transduced with an iNKT-like TCR (Clone_197) found in T1D subjects (Table S3). Transduced CD4 T cells were labeled with CFSE and stimulated with the indicated islet peptides or galactosylceramide (KRN 7000) for 5 days, then analyzed by flow cytometry. Cyan, non-transduced cells that did not express the murine *Trbc* protein (murine *Trbc*⁻-); magenta, transduced cells that that expressed the murine *Trbc* protein (murine *Trbc*⁺).

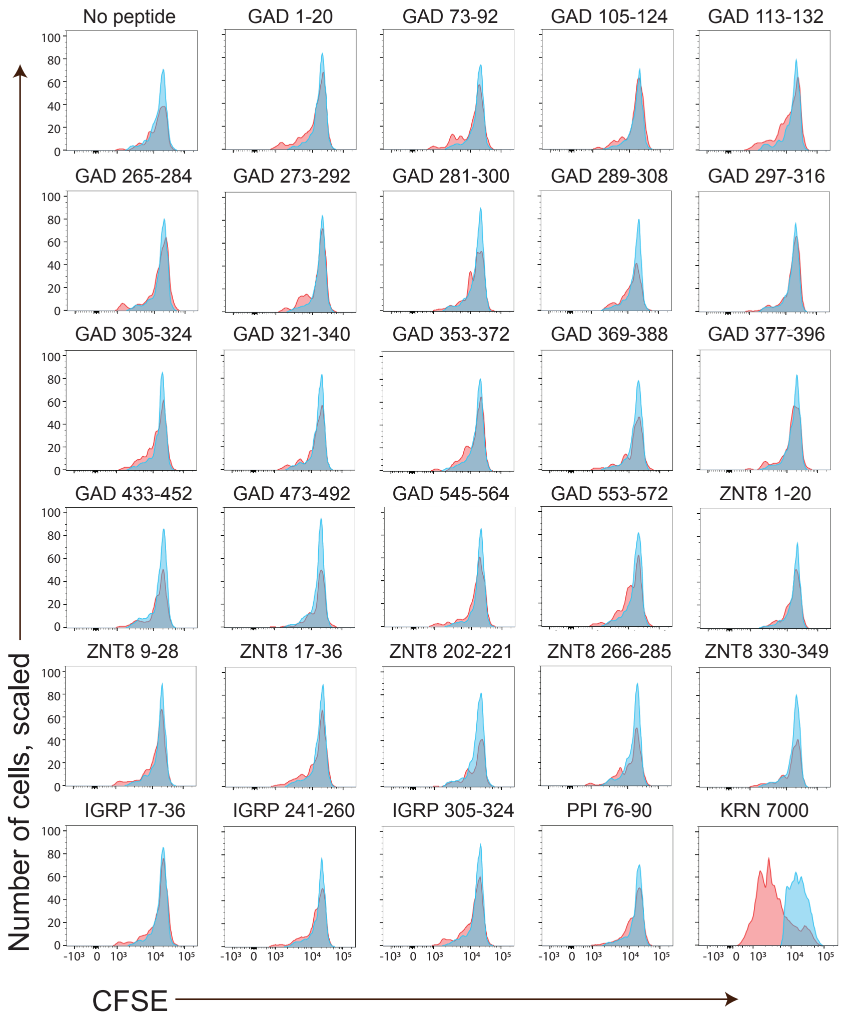

Figure S5. Clonal diversity and persistence of expanded TCRs. Simpson's diversity of TCRs from different disease groups. Filtered junctions (combined *TRA* and *TRB*) from HC, newT1D and T1D subjects (Table S3) (N = 808, 1,784 and 1,481, respectively) were iteratively downsampled to represent 30 cells from each subject prior to calculation of Simpson's diversity. Each symbol represents the median of 1000 iterative downsamples per individual subject. The significance of differences between groups was determined using Wilcoxan signed rank tests. None of the comparisons yielded significant FDR values. Similar results were obtained using individual *TRA* and *TRB* chains.

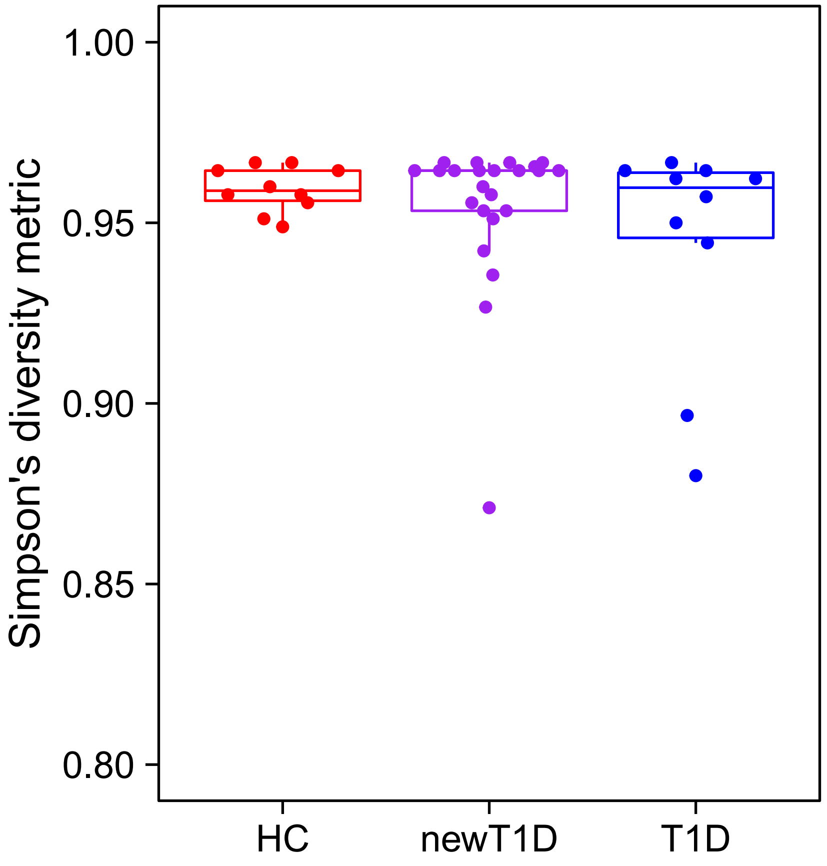

Figure S6. Network graphs show the community landscape of private and public TCRs calculated by *tcrGraph*. Network graphs of public (N = 270) and private (N = 1,130) junctions (Table S3, public/private TCRs) were made using the "stress" layout option of *tcrGraph*. Each graph shows edges (lines) linking nodes (circles) of associated *TRA* (blue) and *TRB* (green) junctions. Node size is proportional to the number of cells containing a particular TCR chain, as indicated in the scale to the right. **A)** Private TCRs. **B)** Public TCRs.

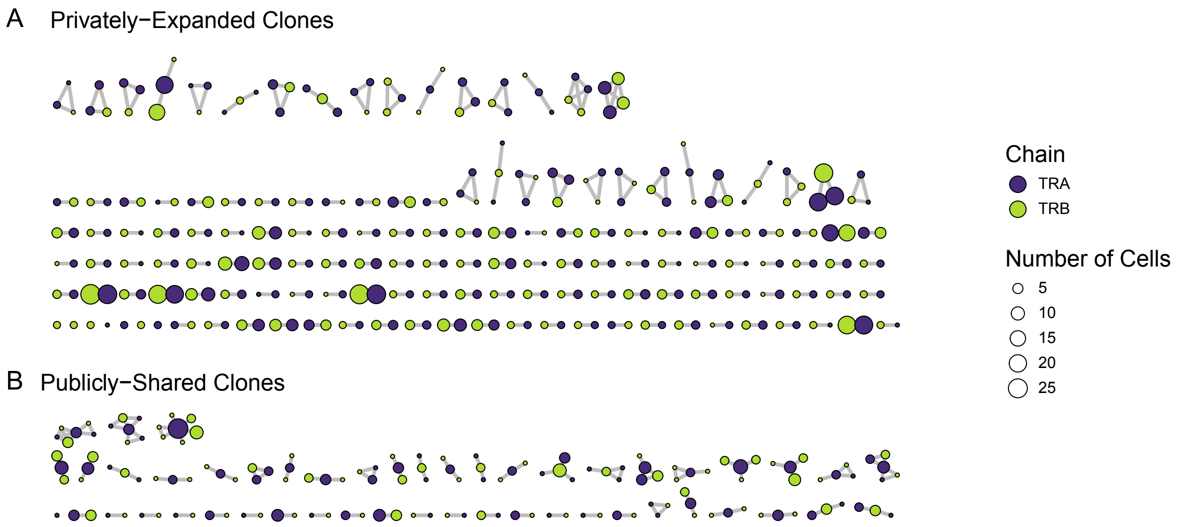

Figure S7. Public TCRs are more likely to have multiple distinct *TRB* junctions sharing an identical *TRA* chain. Shown is a compilation of the predominant combinations of distinct *TRB* and *TRA* junctions identified by *tcrGraph* in public and private TCR clones from network graphs shown in Figure S6.

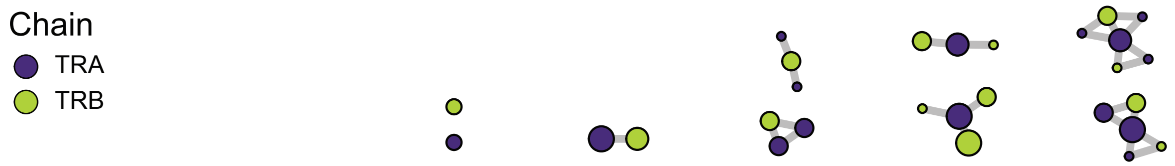

	N clones	Single chain	1 TRA 1 TRB	multiple TRA 1 TRB	1 TRA multiple TRB	multiple TRA multiple TRB
Public	49	1	17	10	16	5
Private	145	8	108	21	6	2
FDR, Fisher's test		4.53e-1	2.30e-6	4.53e-1	2.30e-6	2.00e-2

Figure S8. Public *TRB* junctions did not share more unique *TRA* junctions than private *TRB* junctions. Numbers of *TRA* junctions paired with unique public and private *TRB* junctions were calculated (N = 78 and 147 unique public and private *TRB* junctions, respectively) (Table S3, public/private TCRs). **A)** Combinations of unique *TRB* junctions associated with *TRA* junctions identified by *tcrGraph*. **B)** Tabulation of numbers of *TRA* junctions associated with each unique *TRB* junction. The significance of one versus multiple *TRA* junctions per *TRB* junction in public and private TCRs was assessed by Fisher's exact test. NS, *p*-value >0.05.

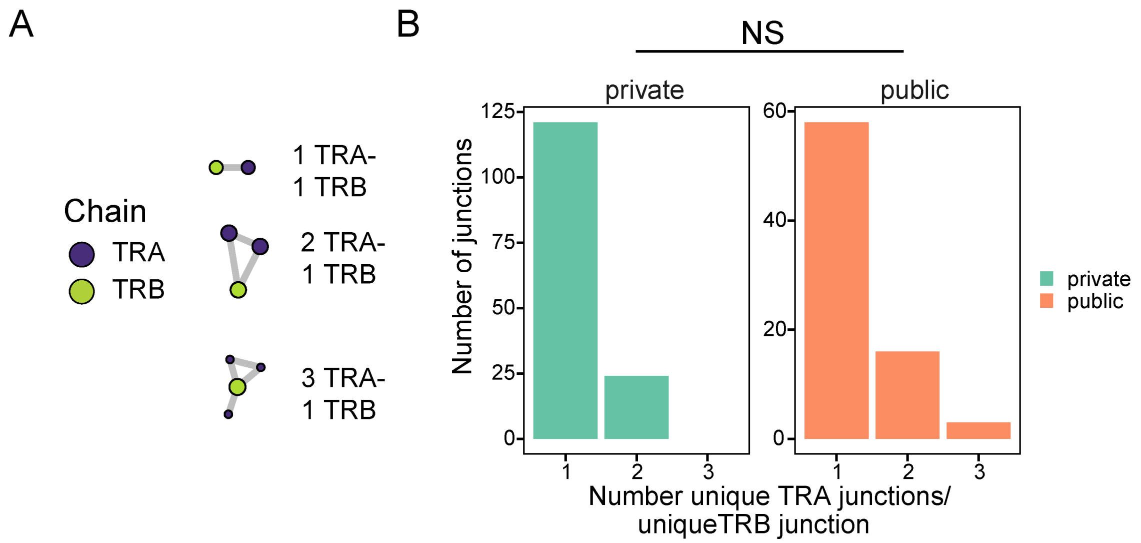

Supplement: Supplemental data [file jciinsight-6-151349-s018.pdf]
